# Bardoxolone conjugation enables targeted protein degradation of BRD4

**DOI:** 10.1038/s41598-020-72491-9

**Published:** 2020-09-23

**Authors:** Bingqi Tong, Mai Luo, Yi Xie, Jessica N. Spradlin, John A. Tallarico, Jeffrey M. McKenna, Markus Schirle, Thomas J. Maimone, Daniel K. Nomura

**Affiliations:** 1 Department of Chemistry, University of California, Berkeley, CA 94720 USA; 2 Novartis-Berkeley Center for Proteomics and Chemistry Technologies, Berkeley, CA USA; 3 Novartis Institutes for BioMedical Research, Cambridge, MA 02139 USA; 4 Departments of Molecular and Cell Biology and Nutritional Sciences and Toxicology, University of California, Berkeley, CA 94720 USA

**Keywords:** Chemical biology, Medicinal chemistry, Organic chemistry, Chemical synthesis, Chemical tools, Natural products, Small molecules

## Abstract

Targeted protein degradation (TPD) has emerged as a powerful tool in drug discovery for the perturbation of protein levels using heterobifunctional small molecules. E3 ligase recruiters remain central to this process yet relatively few have been identified relative to the ~ 600 predicted human E3 ligases. While, initial recruiters have utilized non-covalent chemistry for protein binding, very recently covalent engagement to novel E3’s has proven fruitful in TPD application. Herein we demonstrate efficient proteasome-mediated degradation of BRD4 by a bifunctional small molecule linking the KEAP1-Nrf2 activator bardoxolone to a BRD4 inhibitor JQ1.

## Introduction

**Figure 1:**
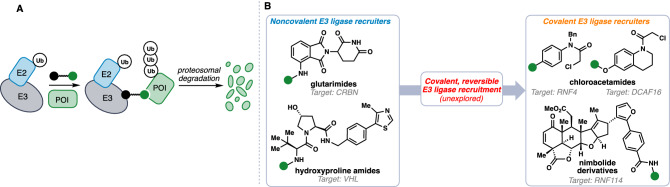
(**A**) Targeted protein degradation using bifunctional small molecules. (**B**) Selected examples of E3 ligase recruiters of varying degrees of covalent engagement.

Targeted protein degradation (TPD) has emerged as a powerful therapeutic modality for drug discovery^1–11^. One strategy available to achieve this therapeutic modality employs heterobifunctional small-molecules known as degraders or proteolysis-targeting chimeras (PROTACs) that are comprised of three constitutive components: a E3 ligase recruiter; a linker; and a ligand to target a protein of interest (POI). By recruiting the E3 ligase to the POI, the resultant PROTAC is able to induce ubiquitination and degradation of the POI in a proteasome-dependent manner (Fig. 1A)^1–11^. While this therapeutic modality has tremendous potential, a major challenge overshadowing the area is that there are only a small number of E3 ligase recruiters that have been identified, this despite there being in excess of 600 predicted E3 ligases. Known and popularized E3 ligase recruiters include thalidomide-type immunomodulatory drugs (IMiDs) that recruit cereblon (CRBN), hydroxyproline-based ligands for the von-Hippel Lindau (VHL) E3 ligase, nutlins that bind to MDM2, and ligands against cIAP (Fig. 1B)^1–15^. While these recruiters bind reversibly to their corresponding E3 ligases, recent studies have revealed that reactive small-molecules that can covalently target E3 ligases can also be used as E3 ligase recruiting modules to potently degrade target proteins in TPD applications. These electrophilic moieties include derivatives of the terpene natural product nimbolide that covalently binds to a disordered cysteine on the E3 ligase RNF114, CCW16 that reacts with a zinc-coordinating cysteine on the E3 ligase RNF4, and KB02 that covalently targets the cullin E3 ligase DCAF16 (Fig. 1B)^16–19^.

Based on the success of covalent E3 ligase recruiters, which have the potential to exploit the vast array of nucleophilic amino acid residues within proteins, we postulated that covalent and reversible E3 ligase recruitment could be a third, underexplored area in PROTAC development (Fig. 1B). As a possible mechanism of action, reversible covalent modification offers the potential for sustained target engagement, while avoiding permanent protein modification—a feature of particular interest given the catalytic nature of PROTACs. While this concept has proven powerful in drug discovery settings^20–25^, we are unaware of its successful employment in E3 ligase recruitment^26,27^. Herein we investigate the small molecule bardoxolone, which possesses a highly reactive—yet reversible—α-cyanoenone hetero Michael acceptor, as the basis for the first protein degrader exploring this concept.

## Results and discussion

**Figure 2:**
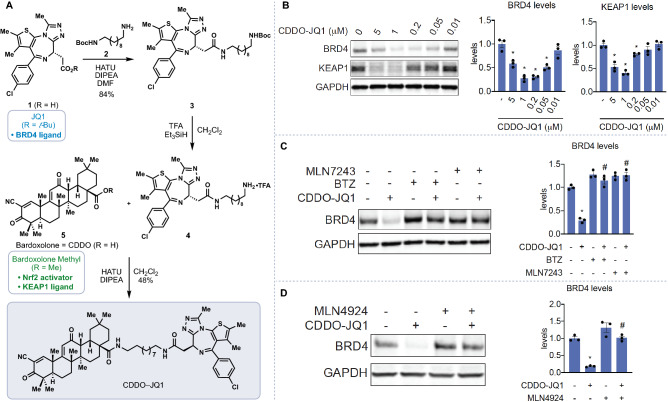
Bardoxolone-based protein degradation. (**A**) Synthesis of CDDO–JQ1. (**B**) Effect of CDDO–JQ1 on BRD4 and KEAP1 levels in 231MFP cells treated with DMSO vehicle or CDDO–JQ1 for 12 h, assessed by Western blotting. (**C**) BRD4 levels in 231MFP cells pre-treated with vehicle, proteasome inhibitor bortezomib (BTZ) (1 μM), or E1 activating enzyme inhibitor MLN7243 (1 μM) for 30 min prior to treatment with vehicle or CDDO–JQ1 (200 nM) for 12 h. (**D**) BRD4 levels in 231MFP cells pre-treated with DMSO vehicle or NEDD8 inhibitor MLN4924 (1 μM) for 30 min prior to treatment with DMSO vehicle or CDDO–JQ1 (200 nM) for 12 h. Blots are representative of n = 3 biological replicates/group. Data in bar graphs is expressed as individual replicate values and average ± sem. Significance shown as *p < 0.05 compared to vehicle-treated control groups and ^#^p < 0.05 compared to CDDO-JQ1-treated groups.

We were intrigued by the possibility of exploiting the triterpene derivative bardoxolone methyl (CDDO-Me) as a covalent, reversible ligase recruiter owing to its reversible interactions with cysteines on the E3 ligase KEAP1 and its highly electron-deficient α-cyanoenone moiety (Fig. 2)^28–30^. To test whether bardoxolone could be used as a novel E3 ligase recruiter, we synthesized a bardoxolone-based PROTAC by linking bardoxolone to the BET family bromodomain protein inhibitor JQ1 (Fig. 2A). The degradation of BET proteins has arisen as a powerful anticancer therapeutic strategy^1,14,31,31^, and the small molecule JQ1 has emerged as a general and robust ligand for use in PROTAC development toward these endeavors^32^. The free acid of JQ1 (**1**) was coupled with Boc-protected amine **2** and the resulting amide (see **3**) converted into TFA salt **4** which was used without further purification^33,34^. Amide bond formation between **4** and CDDO then delivered the bifunctional degrader CDDO–JQ1.

We tested CDDO–JQ1 in the 231MFP human breast cancer cell line and observed dose-responsive degradation of BRD4; at higher concentrations loss of BRD4 degradation was observed, presumably due to the “hook” effect, a phenomenon not unexpected with a highly-reversible, covalently-binding bifunctional molecule (Fig. 2B). Interestingly, we also observed loss of KEAP1 at higher concentrations in cells treated with CDDO–JQ1, a finding that has been reported previously by treating cells with electrophilic stressors (Fig. 2B)^35^. Notably extensive degradation of BRD4 was observed in the 100–200 nM range without any optimization of linker length or composition^36^.

We further demonstrated that the CDDO–JQ1 mediated degradation of BRD4 was attenuated by pre-treatment with the proteasome inhibitor bortezomib as well as the E1 activating enzyme inhibitor MLN7243 (Fig. 2C). Given that KEAP1 belongs to the CUL3 family of E3 ligases that require NEDDylation for activity, we further demonstrated that the BRD4 degradation conferred by CDDO–JQ1 was also significantly attenuated by the NEDD8 activating enzyme inhibitor MLN4924 (Fig. 2D).

**Figure 3:**
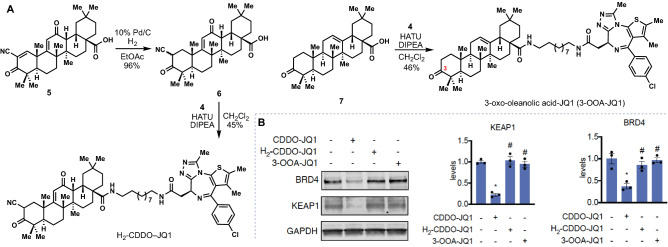
Degraders derived from unreactive variants of CDDO do not support the degradation of BRD4. (**A**) Synthesis of H_2_-CDDO–JQ1 and 3-oxo-oleanoic acid–JQ1 (3-OOA–JQ1). (**B**) Effect of CDDO–JQ1, H_2_-CDDO–JQ1, and 3-OOA–JQ1degraders on BRD4 and KEAP1 levels in 231MFP cells assessed by Western blotting. DMSO vehicle or compounds were treated at 1 μM for 12 h. Blots are representative of n = 3 biological replicates/group. Data in bar graphs is expressed as individual replicate values and average ± sem. Significance shown as *p < 0.05 compared to vehicle-treated control groups and ^#^p < 0.05 compared to CDDO–JQ1-treated groups.

To show that the observed degradation by CDDO–JQ1 was not due to hydrophobic tagging of BRD4 leading to local protein unfolding and subsequent ubiquitination and degradation^38–41^, we synthesized two negative control compounds, both having significantly altered electrophilicity and resultant covalent protein reactivity, but crucially having similar physicochemical properties (Fig. 3A). First, hydrogenation of **5** (H_2_, Pd/C) generated ketone **6** as a mixture of diastereomers and enol tautomers. Coupling of this material with **4** then generated H_2_-CDDO–JQ1. Importantly, H_2_-CDDO–JQ1 did not induce BRD4 degradation in comparison to CDDO–JQ1 (Fig. 3B). Similarly, we prepared 3-oxo-oleanolic acid–JQ1 (3-OOA–JQ1), which possesses no potentially reactive alkenes of any type by coupling **7**^42^ with **4** and found it also did not degrade BRD4 as compared to CDDO–JQ1.

**Figure 4:**
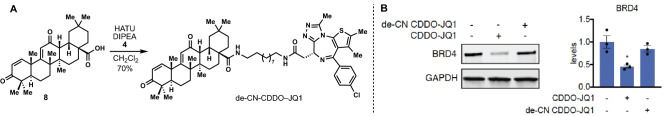
Functional significance of the α-cyanoenone moiety. (**A**) Synthesis of de-CN-CDDO–JQ1. (**B**) Effect of CDDO–JQ1 and de-CN-CDDO–JQ1 on BRD4 and GAPDH levels in 231MFP cells assessed by Western blotting. DMSO vehicle or compounds were treated at 1 μM for 12 h. Blots are representative of n = 3 biological replicates/group. Data in bar graphs is expressed as individual replicate values and average ± sem. Significance shown as *p < 0.05 compared to vehicle-treated control groups and ^#^p < 0.05 compared to CDDO–JQ1-treated groups.

Finally, we prepared the des-cyano variant of CDDO–JQ1 by coupling **8**^43^ and **4** (Fig. 4A), and found that this compound (de-CN-CDDO–JQ1) also does not degrade BRD4 in 231 MFP cells indicating the criticality of the entire α-cyanoenone motif to this process (Fig. 4B). Interestingly, this chemical modification had also previously reduced the activity of bardoxolone methyl ~ 1,000 fold as an anti-inflammatory agent^30^. In general, all three of the changes made to the CDDO portion of CDDO–JQ1 (Figs. 3 and 4) were known to greatly reduce Nrf2 activation in the medicinal chemistry campaigns involving bardoxolone methyl as an anti-inflammatory drug^28–30^.

## Conclusion

In summary, the first bifunctional protein degrader (PROTAC) based on the known KEAP1 ligand bardoxolone (CDDO) is reported. While robust, proteasome-dependent degradation of BRD4 was observed, a number of mechanistic questions remain: firstly, CDDO is not known to bind the Kelch domain of the KEAP1/Cul3 complex, the key area for recognition of Nrf2 and where degrader^44^, and multiple small molecule inhibitors presumably bind^45–48^. While a crystal structure of CDDO bound to Cys-151 of the BTB domain of KEAP1 has been solved, various reports have also posited that multiple cysteines are targeted by this class of compounds during the Nrf2 activation process in cells^49–53^. This raises the question how (or if) a CDDO-based PROTAC can mechanistically induce neosubstrate degradation in a KEAP1-dependent manner. Secondly, while bardoxolone is thought to activate Nrf2 through the targeting of reactive cysteines on KEAP1, it also interacts with additional pharmacological targets, including IKKb which modulates NF-kB signaling^54^, mTOR^55^, and others^55,56^. It should be noted that the direct detection of all proteome-wide targets of CDDO by pulldown studies has proven challenging given the highly reversible nature of its cysteine interactions^30,50,53,54–56^. Thus, we cannot rule out at this moment that the degradation observed here may be due to one (or more) other cullin-family E3 ligases that are targeted by CDDO–JQ1.

Emerging evidence suggests that clinical resistance to PROTACs can occur through rewiring of the cellular E3 ligase machinery^57,58^, thus highlighting the critical need for more and diverse E3 ligase recruiters. Our combined results reported herein strongly implicate E3 ligase involvement and covalent cysteine reactivity in the mechanism of BRD4 degradation by CDDO–JQ1. Future chemoproteomic and genetic studies to map the proteome-wide targets of CDDO–JQ1 will be revealing in further understanding the mechanism of degradation reported herein. Nevertheless, the degradation toolkit has now been expanded to include CDDO as an easily prepared recruiter, and reversible, covalent E3 engagement as a promising concept for future TPD applications.

## Experimental section

### General synthetic methods

Unless otherwise noted, all reactions were performed in flame-dried glassware under a positive pressure of nitrogen or argon. Air- and moisture-sensitive liquids were transferred via syringe. Dry dichloromethane (CH_2_Cl_2_) and *N*,*N*-dimethylformamide (DMF) were obtained by passing these previously degassed solvents through activated alumina columns. Bardoxolone methyl (CDDO-Me) was purchased from Sigma-Aldrich. Oleanolic acid was purchased from Acros Organics. (+)-JQ1 was purchased from Enovation Chemicals. All reagents were used as received from commercial sources, unless stated otherwise. Reactions were monitored by thin layer chromatography (TLC) on TLC silica gel 60 F_254_ glass plates (EMD Millipore) and visualized by UV irradiation and staining with *p*-anisaldehyde, phosphomolybdic acid, or Ninhydrin. Volatile solvents were removed under reduced pressure using a rotary evaporator. Flash column chromatography was performed using Silicycle F60 silica gel (60 Å, 230–400 mesh, 40–63 μm). Proton nuclear magnetic resonance (^1^H NMR) and carbon nuclear magnetic resonance (^13^C NMR) spectra were recorded on Bruker AV-600 and AV-700 spectrometers operating at 600 and 700 MHz for ^1^H NMR, and 151 and 176 MHz for ^13^C NMR (Supplementary Information). Chemical shifts are reported in parts per million (ppm) with respect to the residual solvent signal CDCl_3_ (^1^H NMR: δ 7.26; ^13^C NMR: δ 77.16), CD_2_Cl_2_ (^1^H NMR: δ 5.32; ^13^C NMR: δ 53.84). Peak multiplicities are reported as follows: *s* = singlet, *d* = doublet, *t* = triplet, *dd* = doublet of doublets, *tt* = triplet of triplets, *m* = multiplet, *br* = broad signal. IR spectra were recorded on a Bruker Vertex80 FTIR spectrometer. High-resolution mass spectra (HRMS) were obtained by the QB3/chemistry mass spectrometry facility at the University of California, Berkeley using a Thermo LTQ-FT mass spectrometer with electrospray ionization (ESI) technique.

**Synthesis of compound 3**: To a 10 mL reaction tube was added **1**^33^ (21.7 mg, 0.0541 mmol, 1.0 equiv) and DMF (1.0 mL). DIPEA (37.7 μL, 0.216 mmol, 4.0 equiv) and HATU (22.6 mg, 0.0595 mmol, 1.1 equiv) were added at 0 °C and the reaction mixture was stirred at room temperature for 30 min. The reaction mixture was then cooled back to 0 °C and amine **2**^34^ (29.5 mg, 0.108 mmol, 2.0 equiv) was added. The resulting mixture was allowed to stir at room temperature for 1 h before quenching with saturated *aq.* NH_4_Cl (30 mL). The layers were separated, and the aqueous phase was extracted with EtOAc (3 × 5 mL). The combined organic layer was dried over Na_2_SO_4_ and concentrated *in vacuo*. The residue was purified by column chromatography (3% MeOH/CH_2_Cl_2_) to afford compound **3** (30.0 mg, 84% yield) as a yellow oil: ^1^H NMR (600 MHz, CDCl_3_) δ 7.40 (d, *J* = 8.1 Hz, 2H), 7.32 (d, *J* = 8.2 Hz, 2H), 6.39 (br, 1H), 4.61 (t, *J* = 7.0 Hz, 1H), 4.51 (br, 1H), 3.55 (dd, *J* = 14.0, 7.5 Hz, 1H), 3.39–3.27 (m, 2H), 3.22 (dt, *J* = 13.2, 6.6 Hz, 1H), 3.10 (br, 2H), 2.66 (s, 3H), 2.40 (s, 3H), 1.67 (s, 3H), 1.56–1.42 (m, 4H), 1.44 (s, 9H), 1.33–1.22 (m, 12H); ^13^C NMR (151 MHz, CDCl_3_) δ 170.5, 164.0, 156.1, 155.8, 150.0, 136.9, 136.8, 132.3, 131.1, 130.9, 130.6, 129.9, 128.9, 79.1, 54.7, 40.8, 39.85, 39.77, 30.2, 29.68, 29.61, 29.59, 29.41, 29.40, 28.6, 27.1, 26.9, 14.5, 13.2, 12.0; IR (thin film) vmax 3,347, 2,956, 2,924, 2,853, 1721, 1,453, 1,376, 1,274, 1,176, 1,110, 714 cm^−1^; HRMS (ESI) *calcd.* for [C_34_H_48_O_3_N_6_ClS]^+^ ([M+H]^+^): *m/z* 655.3192, found: 655.3180.

**Synthesis of CDDO–JQ1**: *i.* To a 10 mL reaction tube was added compound **3** (24.3 mg, 0.037 mmol, 1.0 equiv), triethylsilane (12.0 μL, 0.074 mmol, 2.0 equiv) and CH_2_Cl_2_ (0.6 mL). Trifluoroacetic acid (0.2 mL) was added slowly at 0 °C. The reaction mixture was allowed to warm up to room temperature and stirred for 3 h. After completion of the reaction, the mixture was concentrated by rotatory evaporation. The residue was dried under high vacuum for 3 h to provide crude compound **4** as a yellow oil, which was used directly in the next step without further purification.

*ii.* To a 10 mL reaction tube was added CDDO (**5**) (12.7 mg, 0.026 mmol, 1.0 equiv), HATU (10.3 mg, 0.027 mmol, 1.05 equiv), DIPEA (13.5 μL, 0.078 mmol, 3.0 equiv) and CH_2_Cl_2_ (0.4 mL). After stirring at room temperature for 12 h, the reaction was quenched with saturated *aq.* NH_4_Cl and extracted with CH_2_Cl_2_ (3 × 1 mL). The combined organic layer was dried over Na_2_SO_4_ and concentrated *in vacuo*. The resulting activated ester was then transferred into another reaction tube containing the previously prepared crude compound **4** (0.037 mmol, 1.4 equiv). CH_2_Cl_2_ (0.4 mL) and DIPEA (22.5 μL, 0.129 mmol, 5.0 equiv) was added and the reaction mixture was stirred at room temperature for 12 h before quenching with saturated aq. NH_4_Cl. The layers were separated, and the aqueous phase was extracted with CH_2_Cl_2_ (3 × 1 mL). The combined organic layer was dried over Na_2_SO_4_ and concentrated *in vacuo*. The residue was purified by column chromatography (4% MeOH/CH_2_Cl_2_) followed by preparative TLC (5% MeOH/CHCl_3_) to afford degrader **CDDO–JQ1** (12.8 mg, 48% yield) as a colorless oil which slowly solidifies: ^1^H NMR (700 MHz, CD_2_Cl_2_) δ 8.09 (s, 1H), 7.43 (d, *J* = 8.3 Hz, 2H), 7.34 (d, *J* = 8.3 Hz, 2H), 6.49 (t, *J* = 5.9 Hz, 1H), 5.95 (s, 1H), 5.85 (t, *J* = 5.8 Hz, 1H), 4.57 (t, *J* = 6.9 Hz, 1H), 3.42 (dd, *J* = 14.3, 7.2 Hz, 1H), 3.32–3.15 (m, 5H), 3.08 (d, *J* = 4.6 Hz, 1H), 2.89–2.84 (m, 1H), 2.63 (s, 3H), 2.40 (s, 3H), 2.00 (td, *J* = 14.0, 3.9 Hz, 1H), 1.81–1.69 (m, 5H), 1.67 (s, 3H), 1.57–1.47 (m, 6H), 1.46 (s, 3H), 1.45–1.39 (m, 1H), 1.36–1.19 (m, 16H), 1.32 (s, 3H), 1.23 (s, 3H), 1.21–1.15 (m, 1H), 1.14 (s, 3H), 1.00 (s, 3H), 0.96 (s, 3H), 0.89 (s, 3H); ^13^C NMR (151 MHz, CD_2_Cl_2_) δ 199.4, 197.4, 177.0, 170.4, 168.9, 166.6, 164.2, 156.2, 150.4, 137.3, 136.9, 132.8, 131.4, 131.2, 130.7, 130.4, 129.0, 124.4, 115.1, 114.7, 54.9, 49.8, 48.0, 46.7, 46.3, 45.4, 43.0, 42.5, 39.97, 39.89, 39.78, 36.4, 35.0, 34.5, 33.4, 32.19, 32.07, 30.9, 30.2, 30.1, 30.0, 29.83, 29.81, 29.65, 29.65, 28.2, 27.4, 27.3, 27.0, 26.7, 25.0, 23.6, 23.3, 21.8, 18.6, 14.6, 13.2, 12.0; IR (thin film) vmax 3,346, 2,924, 2,852, 1,715, 1,661, 1,593, 1,562, 1,532, 1,465, 1,383, 1,262, 1,091, 1,015, 805 cm^−1^; HRMS (ESI) *calcd* for [C_60_H_79_O_4_N_7_ClS]^+^ ([M+H]^+^): *m/z* 1,028.5597, found: 1,028.5599.

**Synthesis of Compound 6**: To a solution of CDDO (**5**) (5.8 mg, 0.012 mmol, 1.0 equiv) in EtOAc (1 mL) was added 10% Pd/C (5 mg) in one portion. Hydrogen gas was bubbled through the reaction mixture via a long steel needle that was attached to a hydrogen balloon. After 5 min, the needle was lifted above the solvent level and the reaction was stirred for additional 20 min under H_2_ atmosphere. The reaction mixture was filtered through celite, washed with EtOAc (2 mL) and concentrated *in vacuo* to afford compound **6** (5.6 mg, 96% yield) as a white solid, which was pure enough (> 95% by ^1^H NMR) to be used in the next step without further purification. The product exists as a mixture of diastereomers and enol/keto tautomers (all peaks are listed): ^1^H NMR (600 MHz, CDCl_3_) δ 5.77 (s, 0.65H), 5.74 (s, 0.21H), 5.72 (s, 0.14H), 3.98 (dd, *J* = 13.8, 5.4 Hz, 0.21H), 3.93 (dd, *J* = 11.7, 8.1 Hz, 0.14H), 3.06–2.98 (m, 1H), 2.98–2.90 (m, 1H), 2.71–2.59 (m, 0.35H), 2.42 (d, *J* = 15.0 Hz, 0.65H), 2.37 (dd, *J* = 13.5, 8.1 Hz, 0.14H), 2.25 (d, *J* = 15.0 Hz, 0.65H), 2.03 (t, *J* = 13.5 Hz, 0.21H), 1.98–1.16 (m, 24H), 1.15 (s, 0.42H), 1.14 (s, 0.63H), 1.12 (s, 1.95H), 1.05 (s, 0.42 H), 1.00 (s, 4.95H), 0.99 (s, 0.63H), 0.90 (s, 3H); ^13^C NMR (151 MHz, CDCl_3_) δ 206.3, 204.1, 199.7, 199.5, 199.1, 183.2, 182.9, 175.3, 175.1, 173.7, 171.5, 126.4, 124.6, 123.7, 118.7, 117.1, 116.6, 79.2, 51.8, 49.8, 49.72, 49.70, 49.4, 48.6, 48.5, 47.2, 47.13, 47.11, 47.06, 45.7, 45.6, 45.4, 42.9, 42.3, 42.04, 42.00, 41.9, 39.6, 38.9, 38.73, 38.69, 38.2, 37.5, 36.0, 35.9, 35.8, 35.7, 34.6, 34.5, 33.4, 33.1, 33.0, 32.5, 31.59, 31.58, 31.3, 30.80, 30.77, 30.5, 29.8, 28.9, 28.3, 28.2, 28.1, 28.0, 26.9, 25.1, 24.3, 24.1, 23.22, 23.18, 23.16, 23.12, 22.8, 22.7, 22.6, 21.8, 21.71, 21.68, 21.57, 20.01, 19.96, 19.3, 18.94, 18.88; IR (thin film) 2,927, 2,856, 1,722, 1,694, 1,662, 1,466, 1,385, 1,261, 1,241, 1,186, 1,161, 883, 807, 750 cm^-1^; HRMS (ESI) *calcd* for [C_31_H_42_O_4_N]^–^ ([M − H]^–^): *m/z* 492.3119, found: 492.3120.

**Synthesis of H**_**2**_**-CDDO–JQ1**: To a 10 mL reaction tube was added compound **6** (5.6 mg, 0.011 mmol, 1.0 equiv), HATU (4.7 mg, 0.012 mmol, 1.1 equiv), DIPEA (5.9 μL, 0.034 mmol, 3.0 equiv) and CH_2_Cl_2_ (0.4 mL). After stirring at room temperature for 12 h, the reaction was quenched with saturated aq. NH_4_Cl and extracted with CH_2_Cl_2_ (3 × 1 mL). The combined organic layer was dried over Na_2_SO_4_ and concentrated *in vacuo*. The resulting activated ester was then transferred into another reaction tube containing the previously prepared crude compound **4** (0.017 mmol, 1.5 equiv). CH_2_Cl_2_ (0.4 mL) and DIPEA (9.8 μL, 0.056 mmol, 5.0 equiv) was added and the reaction mixture was stirred at room temperature for 12 h before quenching with saturated *aq.* NH_4_Cl. The layers were separated, and the aqueous phase was extracted with CH_2_Cl_2_ (3 × 1 mL). The combined organic layer was dried over Na_2_SO_4_ and concentrated *in vacuo*. The residue was purified by preparative TLC (5% MeOH/CHCl_3_) to afford **H**_**2**_**-CDDO–JQ1** (5.2 mg, 45% yield) as a colorless oil which slowly solidifies (mixture of diasteromers and enol/keto tautomers): ^1^H NMR (600 MHz, CD_2_Cl_2_) δ 7.43 (d, *J* = 8.4 Hz, 2H), 7.35 (d, *J* = 8.3 Hz, 2H), 6.47 (t, *J* = 5.8 Hz, 0.6H), 6.47 (t, *J* = 5.8 Hz, 0.4H), 5.91–5.80 (m, 1H), ), 5.74 (s, 0.4H), 5.72 (s, 0.4H), 5.69 (s, 0.2H), 4.62–4.54 (m, 1H), 4.04 (dd, *J* = 14.1, 5.1 Hz, 0.4H), 3.96 (dd, *J* = 12.0, 7.8 Hz, 0.2H), 3.49–3.39 (m, 1H), 3.34–3.14 (m, 5H), 3.07–2.99 (m, 1H), 2.89–2.81 (m, 1H), 2.70–1.18 (m, 48H), 1.18–1.14 (m, 3H), 1.13–1.10 (m, 3H), 1.05–0.95 (m, 6H), 0.92–0.87 (m, 3H); ^13^C NMR (151 MHz, CD_2_Cl_2_) δ 207.3, 205.0, 200.0, 199.6, 177.10, 177.06, 175.9, 175.5, 174.2, 171.4, 170.43, 170.35, 164.24, 164.18, 156.15, 156.10, 150.5, 150.4, 137.29, 137.26, 136.92, 136.89, 132.8, 132.7, 131.5, 131.4, 131.17, 131.15, 130.8, 130.7, 130.4, 129.0, 126.6, 124.6, 123.7, 119.1, 117.7, 117.3, 80.1, 54.92, 54.90, 52.0, 49.8, 49.66, 49.64, 49.4, 48.8, 47.3, 46.8, 46.75, 46.73, 46.1, 46.0, 45.8, 43.3, 42.7, 42.25, 42.23, 42.1, 39.95, 39.89, 39.88, 39.8, 39.10, 39.05, 38.99, 38.8, 38.0, 36.6, 36.5, 36.4, 36.1, 35.02, 34.99, 34.6, 33.45, 33.43, 32.8, 32.3, 32.2, 31.6, 30.95, 30.92, 30.8, 30.30, 30.26, 30.15, 30.10, 30.05, 30.02, 29.99, 29.91, 29.83, 29.80, 29.73, 29.67, 29.64, 28.9, 28.3, 28.24, 28.16, 27.9, 27.6, 27.43, 27.40, 27.3, 27.0, 24.9, 24.6, 24.3, 23.65, 23.54, 23.51, 23.48, 23.33, 23.29, 23.2, 21.9, 21.80, 21.75, 21.68, 20.0, 19.9, 19.6, 19.22, 19.15, 14.6, 13.2, 12.0; IR (thin film) vmax 3,345, 2,924, 2,853, 1,728, 1,660, 1,595, 1532, 1,465, 1,417, 1,382, 1,091, 1,015, 840, 805, 721 cm^−1^; HRMS (ESI) *calcd.* for [C_60_H_81_O_4_N_7_ClS]^+^ ([M + H]^+^): *m/z* 1,030.5754, found: 1,030.5744.

**3-Oxo-oleanolic acid–JQ1 (3-OOA–JQ1)**: to a 10 mL reaction tube was added compound **7**^42^ (9.3 mg, 0.0204 mmol, 1.0 equiv) and CH_2_Cl_2_ (1.0 mL). The resulting solution was cooled to 0 °C, and DIPEA (14.3 μL, 0.0818 mmol, 4.0 equiv) and HATU (8.5 mg, 0.0225 mmol, 1.1 equiv) were added. The reaction mixture was allowed to stir at room temperature for 30 min before quenching with saturated aq. NH_4_Cl. The layers were separated, and the aqueous phase was extracted with CH_2_Cl_2_ (3 × 1 mL). The combined organic layer was dried over Na_2_SO_4_ and concentrated *in vacuo*. The resulting activated ester was then transferred into another reaction tube containing previously prepared crude compound **4** (17.0 mg, 0.0307 mmol, 1.5 equiv), DIPEA (35.5 μL, 0.204 mmol, 10.0 equiv) and CH_2_Cl_2_ (1.0 mL). The reaction mixture was allowed to stir at room temperature for 12 h before quenching with saturated aq. NH_4_Cl. The layers were separated, and the aqueous phase was extracted with CH_2_Cl_2_ (3 × 1 mL). The combined organic layer was dried over Na_2_SO_4_ and concentrated *in vacuo*. The residue was purified by preparative TLC (6% MeOH/CH_2_Cl_2_, developed twice) to afford **3-oxo-oleanolic acid–JQ1** (9.3 mg, 46% yield) as a white solid: ^1^H NMR (600 MHz, CDCl_3_) δ 7.42–7.38 (m, 2H), 7.35–7.31 (m, 2H), 6.36 (t, *J* = 5.7 Hz, 1H), 5.88 (t, *J* = 5.5 Hz, 1H), 5.39 (t, *J* = 3.6 Hz, 1H), 4.60 (dd, *J* = 7.6, 6.3 Hz, 1H), 3.55 (dd, *J* = 14.1, 7.7 Hz, 1H), 3.39–3.28 (m, 3H), 3.21 (dq, *J* = 13.2, 7.0 Hz, 1H), 3.06–2.97 (m, 1H), 2.67 (s, 3H), 2.59–2.50 (m, 2H), 2.40 (d, *J* = 0.8 Hz, 3H), 2.37 (ddd, *J* = 15.9, 6.8, 3.6 Hz, 1H), 2.03–1.92 (m, 3H), 1.89 (ddd, *J* = 13.2, 7.3, 3.7 Hz, 1H), 1.77 (t, *J* = 13.3 Hz, 1H), 1.74–1.64 (m, 3H), 1.67 (s, 3H), 1.61–1.38 (m, 12H), 1.36–1.18 (m, 16H), 1.17 (s, 3H), 1.09 (s, 3H), 1.06 (s, 3H), 1.05 (s, 3H), 0.91 (s, 3H), 0.91 (s, 3H), 0.83 (s, 3H); ^13^C NMR (151 MHz, CDCl_3_) δ 217.7, 178.1, 170.5, 164.0, 155.8, 150.0, 145.4, 137.0, 136.8, 132.3, 131.1, 131.0, 130.6, 130.0, 128.9, 122.5, 55.4, 54.7, 47.6, 47.0, 46.9, 46.4, 42.6, 42.4, 39.86, 39.82, 39.6, 39.5, 39.3, 36.9, 34.32, 34.30, 33.2, 32.7, 32.1, 30.9, 29.70, 29.70, 29.64, 29.52, 29.46, 29.43, 27.5, 27.3, 27.1, 26.6, 25.8, 23.9, 23.8, 23.7, 21.6, 19.7, 17.0, 15.2, 14.5, 13.2, 12.0. IR (thin film) vmax 3,346, 2,925, 2,854, 1,705, 1,654, 1593, 1532, 1,457, 1,419, 1,381, 1,274, 1,176, 1,110, 1,091, 1,014, 839, 805, 749, 714 cm^-1^; HRMS (ESI) *calcd* for [C_59_H_84_O_3_N_6_ClS]^+^ ([M + H]^+^): *m/z* 991.6009, found: 991.6005.

**De-CN-CDDO–JQ1**: To a 10 mL reaction tube was added compound **8**^43^ (2.4 mg, 0.0051 mmol, 1.0 equiv), HATU (2.2 mg, 0.0057 mmol, 1.1 equiv), DIPEA (2.6 μL, 0.015 mmol, 3.0 equiv) and CH_2_Cl_2_ (0.3 mL). After stirring at room temperature for 12 h, the reaction was quenched with saturated *aq.* NH_4_Cl and extracted with CH_2_Cl_2_ (3 × 1 mL). The combined organic layer was dried over Na_2_SO_4_ and concentrated *in vacuo*. The resulting activated ester was then transferred into another reaction tube containing the previously prepared crude compound **4** (0.0076 mmol, 1.5 equiv). CH_2_Cl_2_ (0.4 mL) and DIPEA (2.6 μL, 0.015 mmol, 3.0 equiv) was added and the reaction mixture was stirred at room temperature for 12 h before quenching with saturated *aq.* NH_4_Cl. The layers were separated, and the aqueous phase was extracted with CH_2_Cl_2_ (3 × 1 mL). The combined organic layer was dried over Na_2_SO_4_ and concentrated *in vacuo*. The residue was purified by preparative TLC (5% MeOH/CHCl_3_) to afford **De-CN-CDDO–JQ1** (3.6 mg, 70% yield) as a colorless oil which slowly solidifies: ^1^H NMR (600 MHz, CD_2_Cl_2_) δ 7.43 (d, *J* = 8.3 Hz, 2H), 7.38–7.31 (m, 3H), 6.49 (t, *J* = 5.9 Hz, 1H), 5.97 (s, 1H), 5.93–5.82 (m, 2H), 4.56 (t, *J* = 6.9 Hz, 1H), 3.42 (dd, *J* = 14.3, 7.2 Hz, 1H), 3.32–3.14 (m, 5H), 3.03 (d, *J* = 4.6 Hz, 1H), 2.91–2.84 (m, 1H), 2.63 (s, 3H), 2.40 (s, 3H), 2.00 (td, *J* = 13.9, 3.9 Hz, 1H), 1.81–1.69 (m, 5H), 1.68 (s, 3H), 1.56–1.42 (m, 7H), 1.40 (s, 3H), 1.38–1.32 (m, 2H), 1.31 (s, 3H), 1.30–1.24 (m, 14H), 1.23–1.17 (m, 2H), 1.15 (s, 3H), 1.08 (s, 3H), 1.00 (s, 3H), 0.96 (s, 3H), 0.89 (s, 3H); ^13^C NMR (151 MHz, CD_2_Cl_2_) δ 203.6, 200.0, 177.1, 172.0, 170.5, 164.2, 156.2, 155.2, 150.4, 137.3, 136.9, 132.8, 131.4, 131.2, 130.7, 130.3, 129.0, 126.0, 123.8, 54.9, 49.7, 48.6, 46.8, 46.1, 45.0, 42.4, 42.2, 40.0, 39.9, 39.7, 36.4, 35.0, 34.6, 33.4, 32.5, 32.2, 30.9, 30.3, 30.1, 30.0, 29.83, 29.80, 29.6, 28.2, 27.4, 27.29, 27.25, 26.9, 25.0, 23.6, 23.3, 21.83, 21.80, 18.7, 14.6, 13.2, 12.0; IR (thin film) vmax 3,366, 2,925, 2,854, 1,659, 1,596, 1,535, 1,462, 1,382, 1,263, 1,093, 1,016, 846, 801, 605 cm^−1^; HRMS (ESI) *calcd.* for [C_59_H_80_O_4_N_6_ClS]^+^ ([M + H]^+^): *m/z* 1,003.5645, found: 1,003.5623 (Supplementary Information).

### General biological methods

#### Materials

Primary antibodies to BRD4 (Abcam, Ab128874), GAPDH (Proteintech Group Inc., 60004-1-Ig), and KEAP1 (Cell Signaling Technologies, D6B12) were obtained from commercial sources and dilutions were prepared according to manufacturer recommendations. Anti-rabbit and anti-mouse secondary antibodies were purchased from Licor (IRDye 800CW Goat anti-Rabbit IgG Secondary Antibody and IRDye 700CW Goat anti-Mouse IgG Secondary Antibody). The commercially available inhibitors bortezomib (Alfa Aesar J60378MA), MLN7243 (SelleckChem S8341) and MLN4924 (SelleckChem S7109) were purchased as solids and DMSO solutions were prepared at the appropriate concentrations.

#### Cell culture

231MFP cells were obtained from Prof. B. Cravatt (Scripps) and were generated from explanted tumor xenografts of MDA-MB-231 cells as previously described^59^. They were cultured in L15 medium containing 10% (v/v) fetal bovine serum (FBS), maintained at 37 °C with 0% CO_2_.

#### Cell-based degrader assays and western blotting

For assaying degrader activity, cells were seeded (500,000 for 231MFP cells) into 6 cm tissue culture plates (Corning) in 2.0–2.5 mL of media and allowed to adhere overnight. The following morning, media was replaced with complete media containing the desired concentration of CDDO–JQ1 (or related CDDO-based degrader) diluted from a 1,000 × stock in DMSO. For rescue studies, the cells were pre-treated with proteasome inhibitor (bortezomib, 1 μM), E1 inhibitor (MLN7243, 1 μM) or NEDDylation inhibitor (MLN4924, 1 μM) for 30 min. Cells were subsequently treated with vehicle DMSO or degraders for 12 h. To harvest cells, media was aspirated and cells were washed with 500 μL PBS then 100 µL Radioimmunoprecipitation assay buffer (RIPA buffer) was added to each well and incubated 5 min on ice before scraping and transferring to Eppendorf tubes. The collected cells were vortexed vigorously in the lysis buffer and allowed to sit on ice for 5 additional min before cellular debris was pelleted by spinning at maximum speed for 10 min at 4 °C. Supernatant was transferred to new tubes and total protein was normalized by Pierce BCA Protein Assay. Samples were denatured by addition of 4 × Laemmli’s Loading dye and 30 µg of protein was loaded onto 4–20% TGX Precast gels (BioRad). After gel electrophoresis, proteins were transferred to a nitrocellulose membrane using semi-dry transfer on a Trans-Blot Turbo (BioRad) over 7 min. The membrane was then incubated for 1 h in 5% bovine serum albumin (BSA) in tris-buffered saline containing Tween 20 (TBST) before incubation with the corresponding primary antibody overnight at 4 °C. The membranes were washed in TBST before a 1 h room temperature incubation with secondary antibodies. After a final set of washes, blots were imaged on a LiCor CLX imager and band intensities were quantified using ImageJ software (Supplementary Information).

## Supplementary information

Supplementary Figures.
