# Supplementary material for: Bardoxolone conjugation enables targeted protein degradation of BRD4
Source: Sci Rep. 2020 Sep 23;10:15543. doi: 10.1038/s41598-020-72491-9 (PMC7511954; doi:10.1038/s41598-020-72491-9)

Bardoxolone Conjugation Enables Targeted Protein Degradation of BRD4

Supplementary Information

Bingqi Tong,[#,a,b] Mai Luo,[#,a,b] Yi Xie,[a,b] Jessica N. Spradlin,[a,b] John A. Tallarico,[b,c] Jeffrey M. McKenna,[b,c] Markus Schirle,[b,c] Thomas J. Maimone,[*,a,b] and Daniel K. Nomura[*a,b,d]

[a] Department of Chemistry, University of California, Berkeley, Berkeley, CA, 94720
[b] Novartis-Berkeley Center for Proteomics and Chemistry Technologies
[c] Novartis Institutes for BioMedical Research, Cambridge, MA, 02139
[d] Departments of Molecular and Cell Biology and Nutritional Sciences and Toxicology, University of California, Berkeley, Berkeley, CA, 94720
[#] these authors contributed equally
* Correspondence to: maimone@berkeley.edu or dnomura@berkeley.edu

Table of Contents:

Figure S1. ^{1}H NMR of compound **3**……………………………………………………………S3

Figure S2. ^{13}C NMR of compound **3**……………………………………………………………S4

Figure S3. ^{1}H NMR of CDDO–JQ1……………………………………………………………S5

Figure S4. ^{13}C NMR of CDDO–JQ1……………………………………………………………S6

Figure S5. ^{1}H NMR of compound **6**……………………………………………………………S7

Figure S6. ^{13}C NMR of compound **6**……………………………………………………………S8

Figure S7. ^{1}H NMR of H_2-CDDO–JQ1……………………………………………………………S9

Figure S8. ^{13}C NMR of H_2-CDDO–JQ1……………………………………………………………S10

Figure S9. ^{1}H NMR of 3-oxo-oleanolic acid–JQ1……………………………………………………S11

Figure S10. ^{13}C NMR of 3-oxo-oleanolic acid–JQ1……………………………………………………S12

Figure S11. ^{1}H NMR of de-CN-CDDO–JQ1……………………………………………………S13

Figure S12. ^{13}C NMR of de-CN-CDDO–JQ1……………………………………………………S14

Figure S13. Full length and replicate blots used for manuscript **Figure 2B**…………...………………..S15

Figure S14. Full length and replicate blots used for manuscript **Figure 2C**…………...………………..S16

Figure S15. Full length and replicate blots used for manuscript **Figure 2D**…………...………………..S17

Figure S16. Full length and replicate blots used for manuscript **Figure 3B**…………...………………..S18

Figure S17. Full length and replicate blots used for manuscript **Figure 4B**…………...………………..S19

Figure S1. ^{1}H NMR of compound **3**

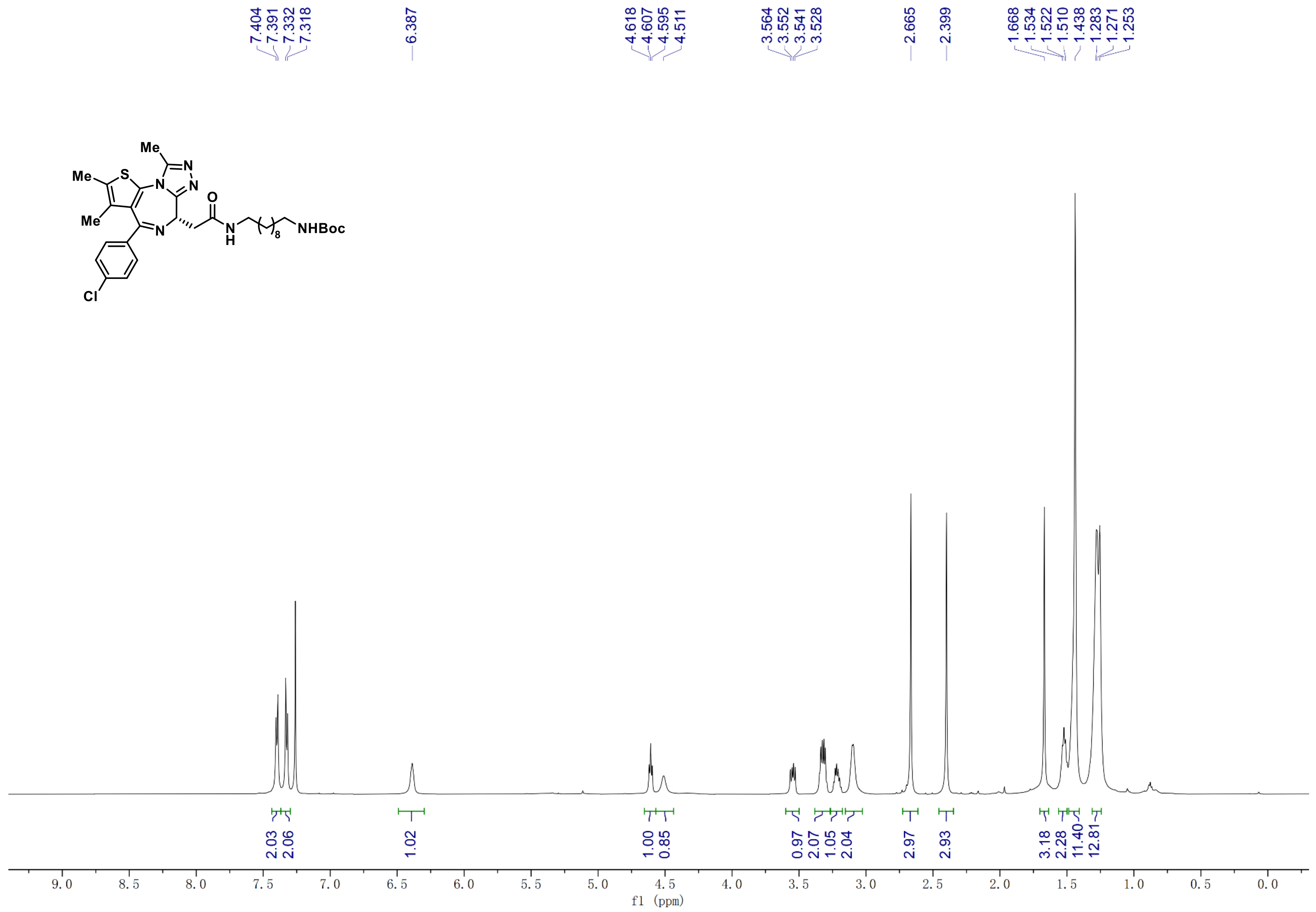

Figure S2. ^{13}C NMR of compound **3**

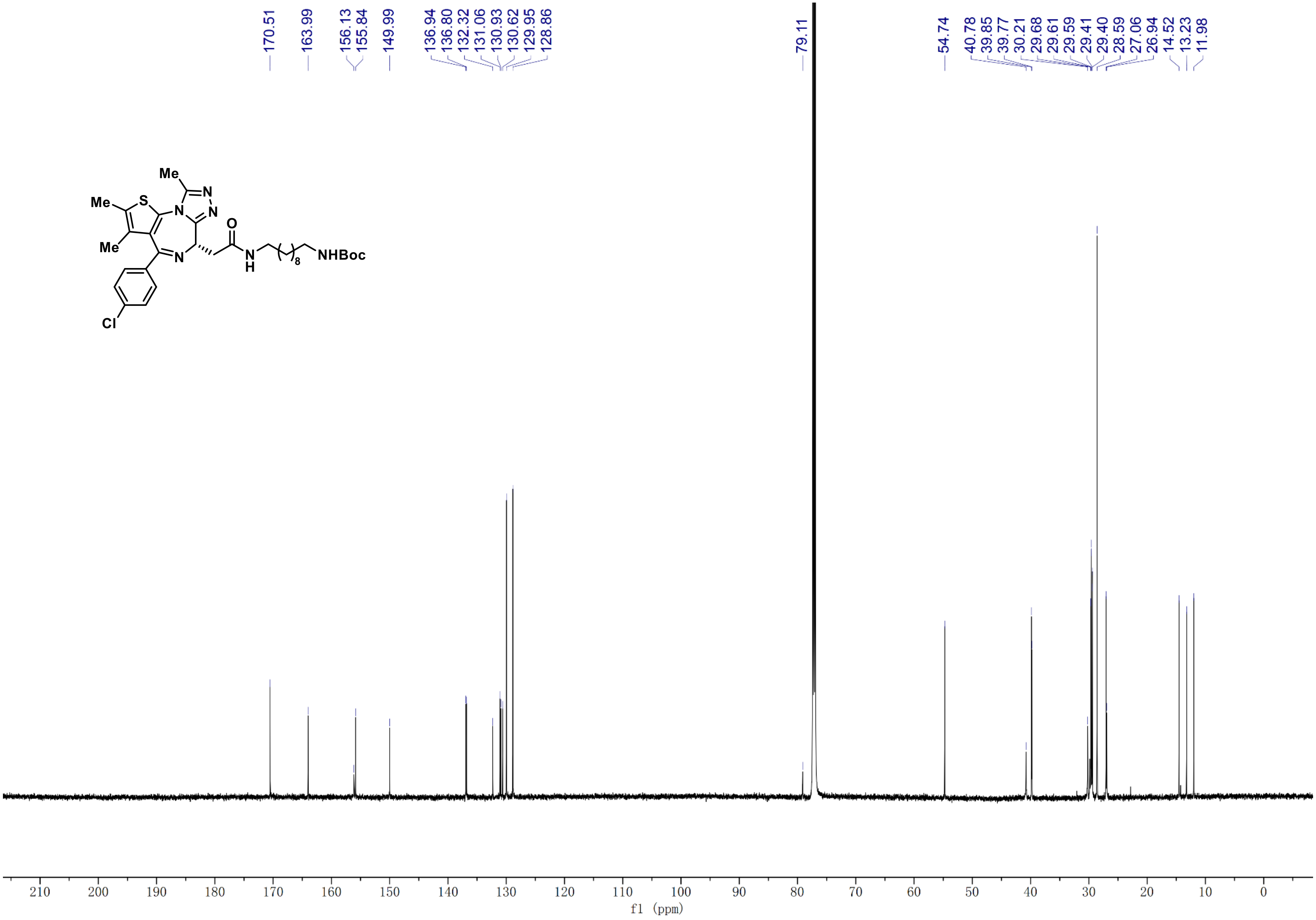

Figure S3. ^{1}H NMR of CDDO–JQ1

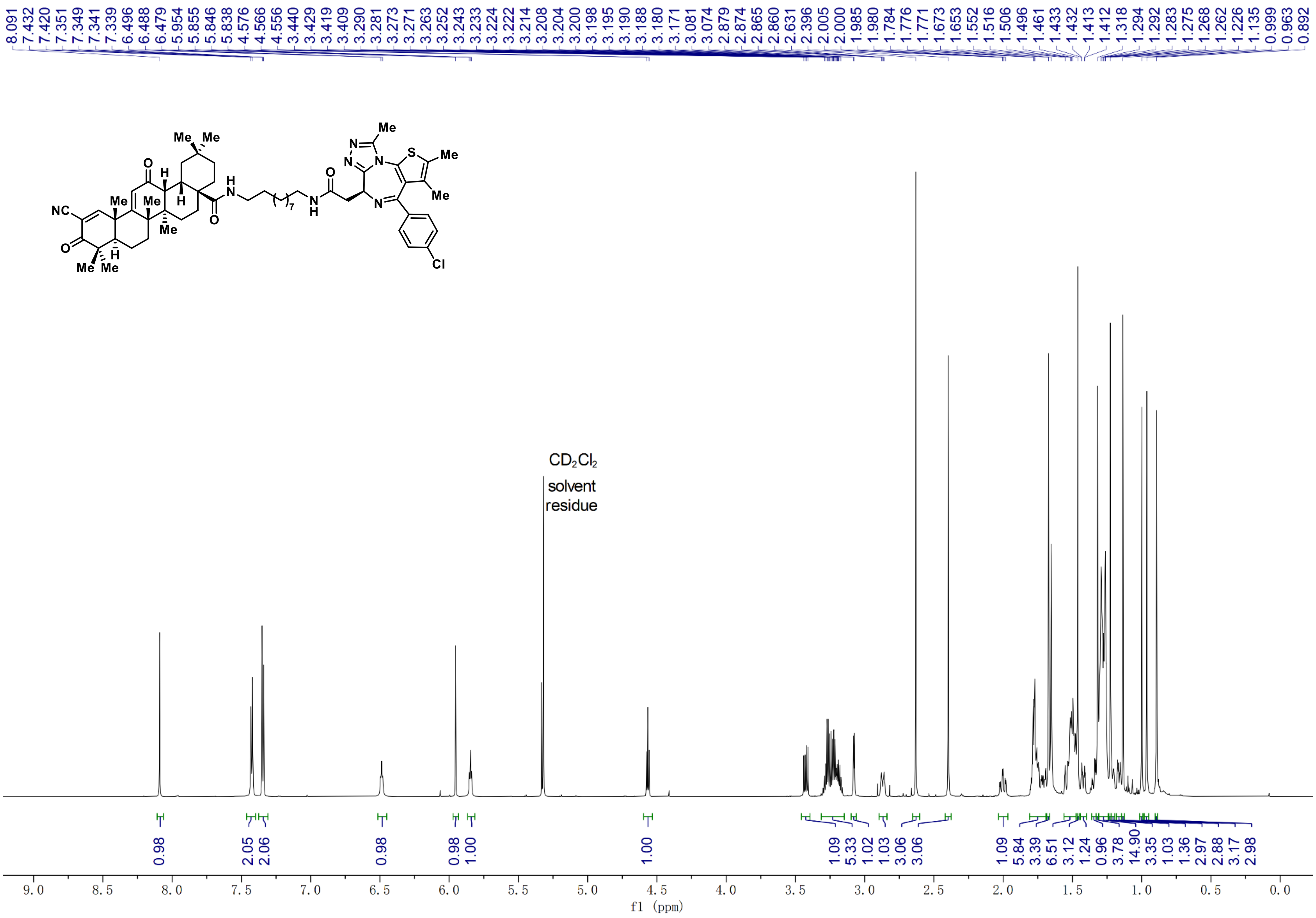

Figure S4. ^{13}C NMR of CDDO–JQ1

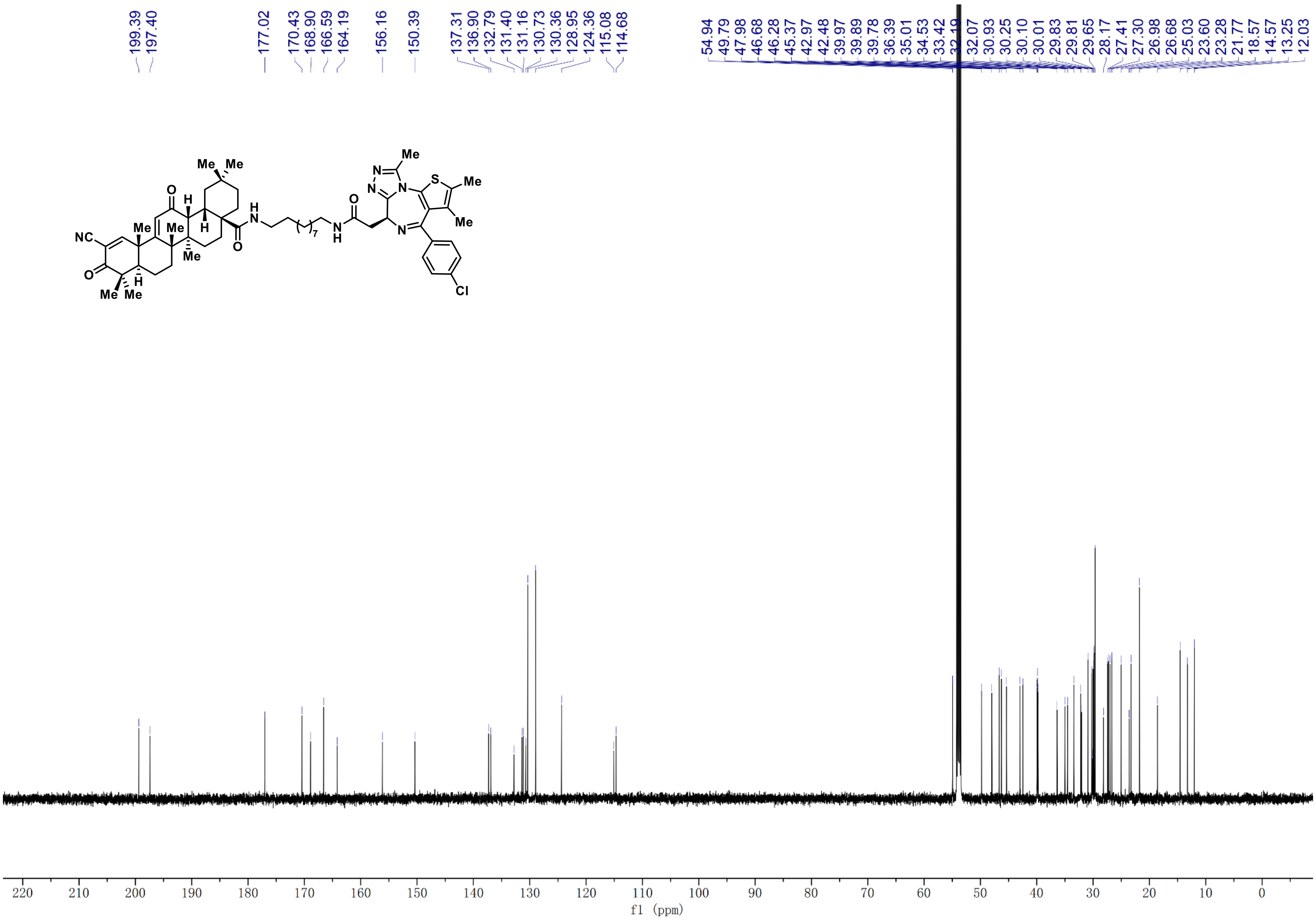

Figure S5. ^{1}H NMR of compound **6**

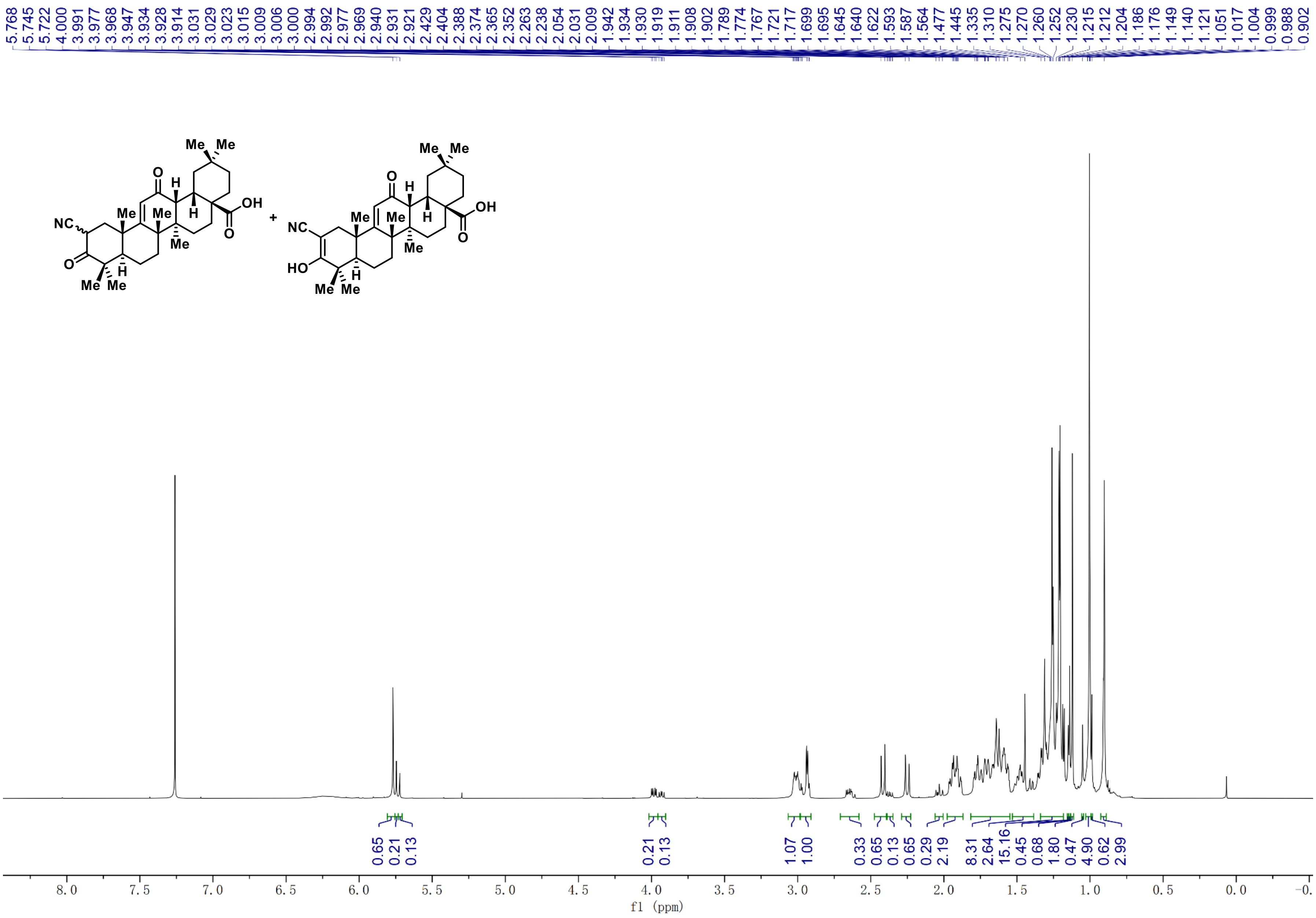

Figure S6. ^{13}C NMR of compound **6**

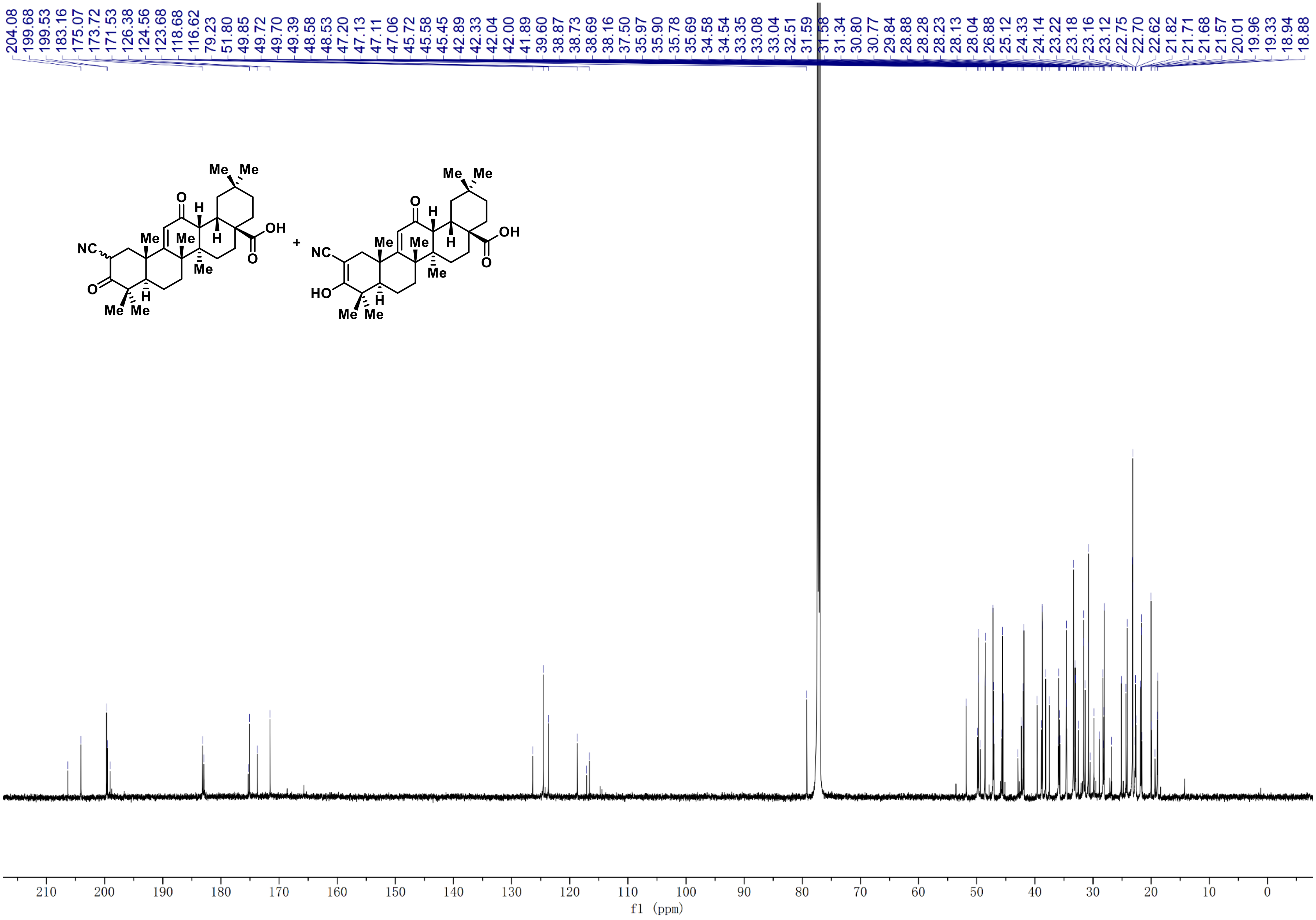

Figure S7. ^{1}H NMR of H_2-CDDO–JQ1

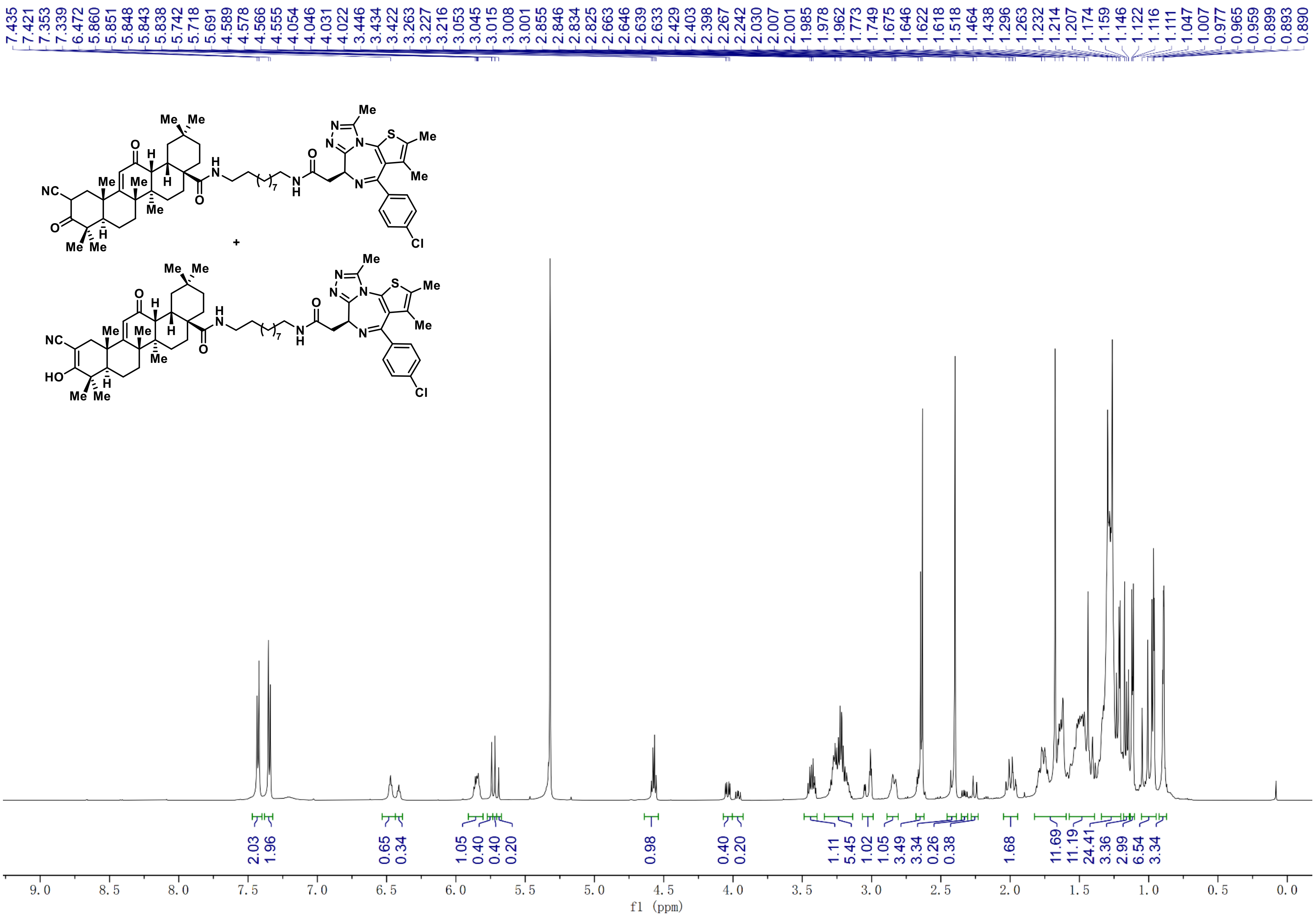

Figure S8. ^{13}C NMR of H_2-CDDO–JQ1

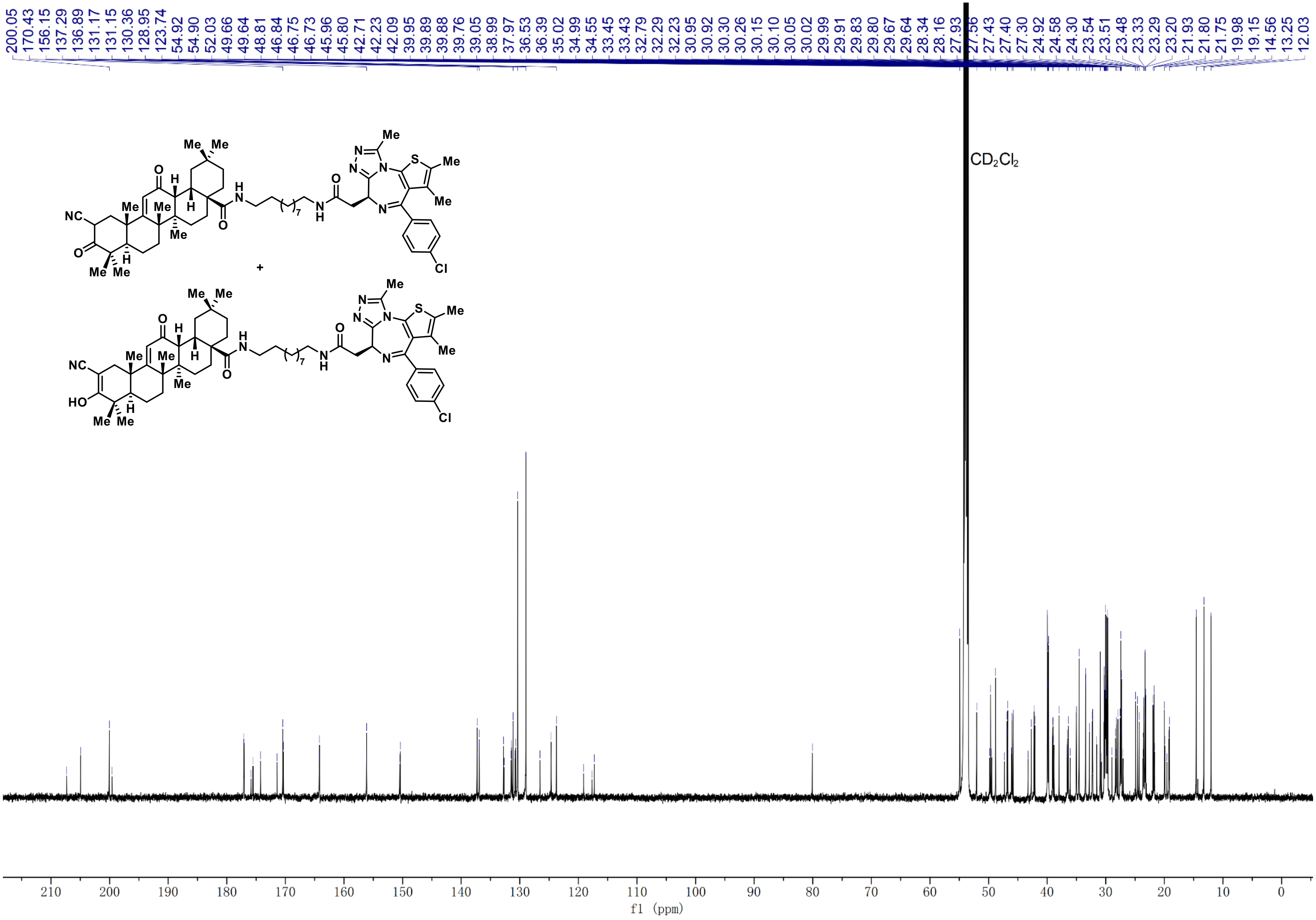

Figure S9. ^{1}H NMR of 3-oxo-oleanolic acid–JQ1

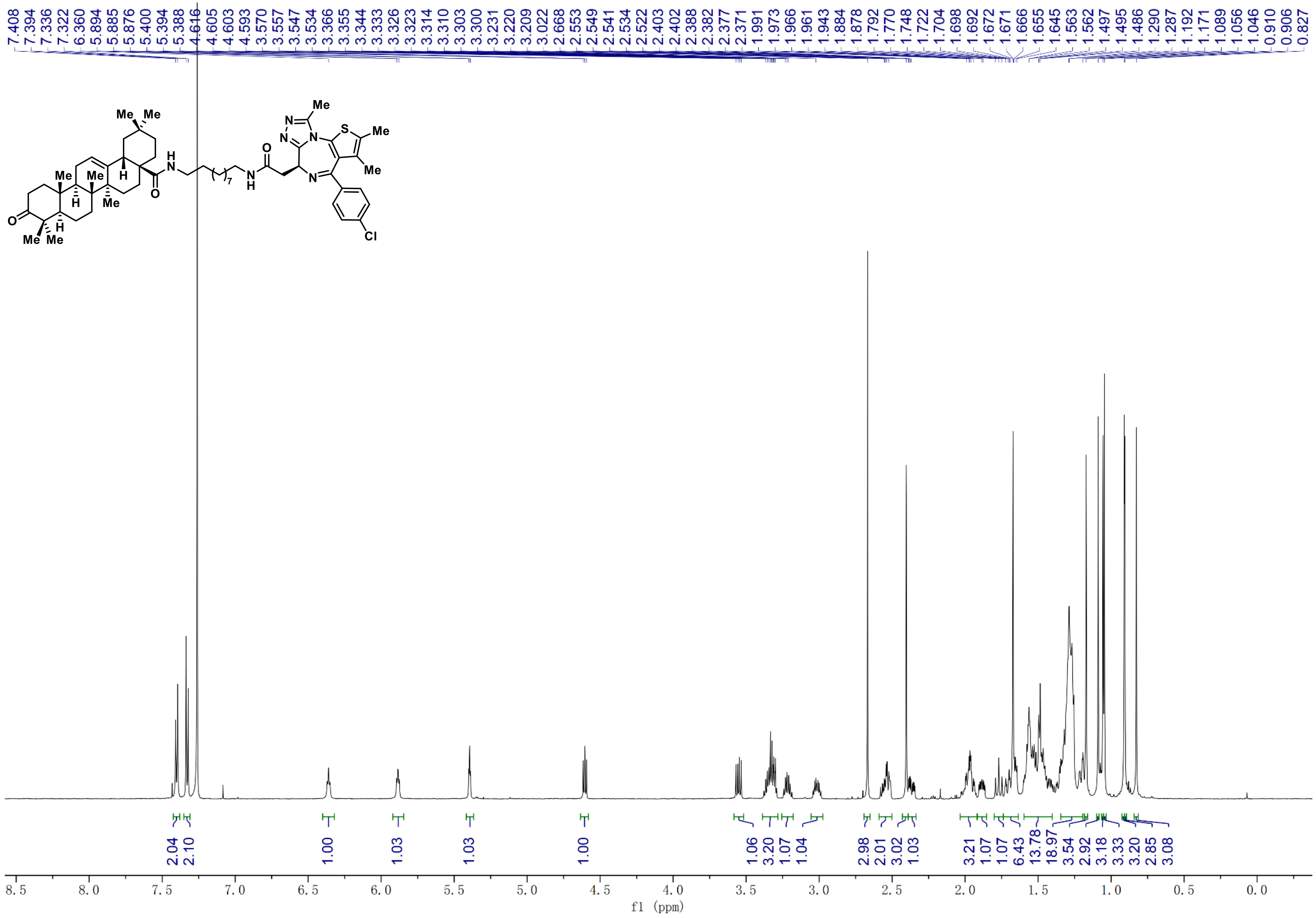

Figure S10. ^{13}C NMR of 3-oxo-oleanolic acid–JQ1

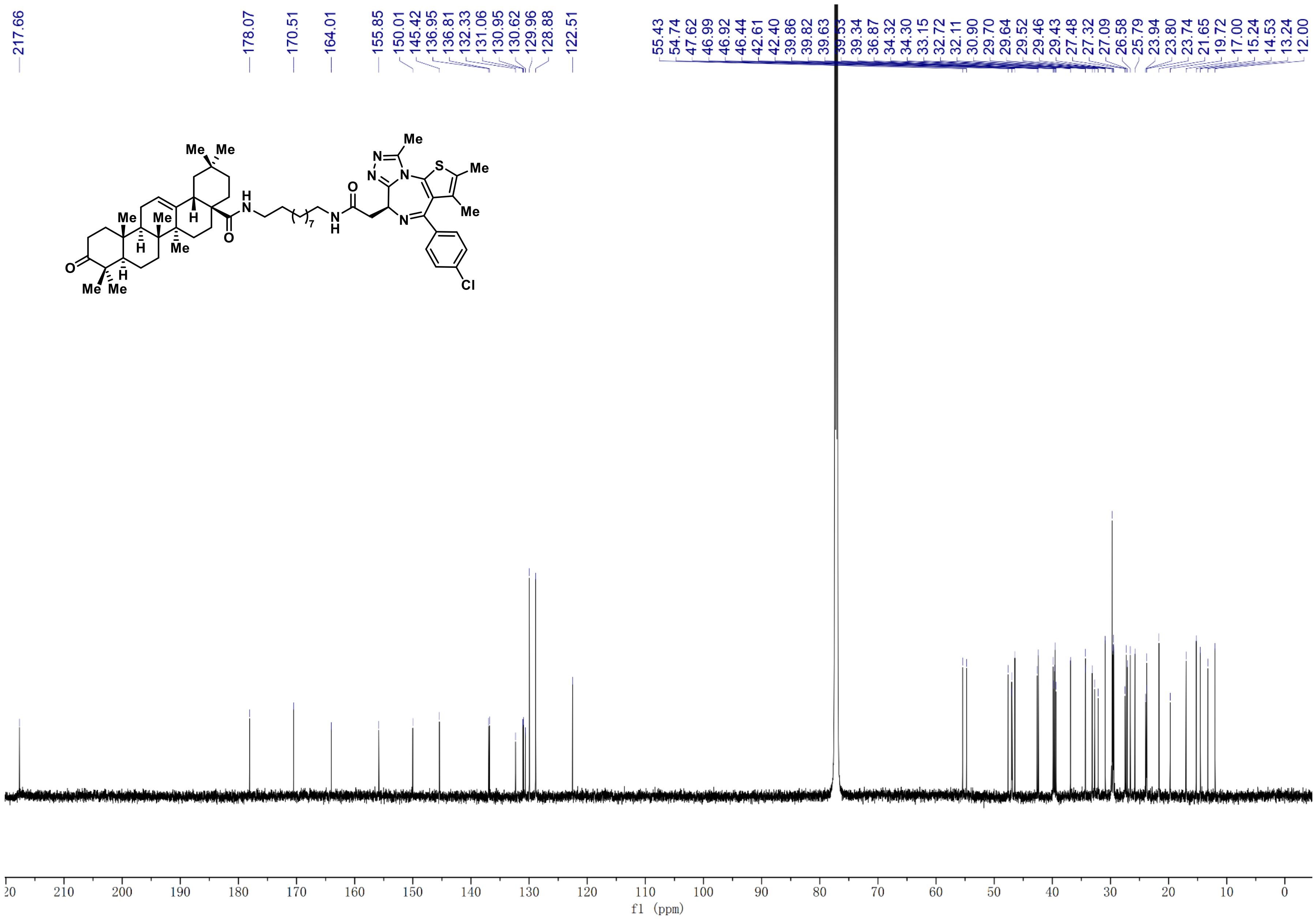

Figure S11. ^{1}H NMR of de-CN-CDDO–JQ1

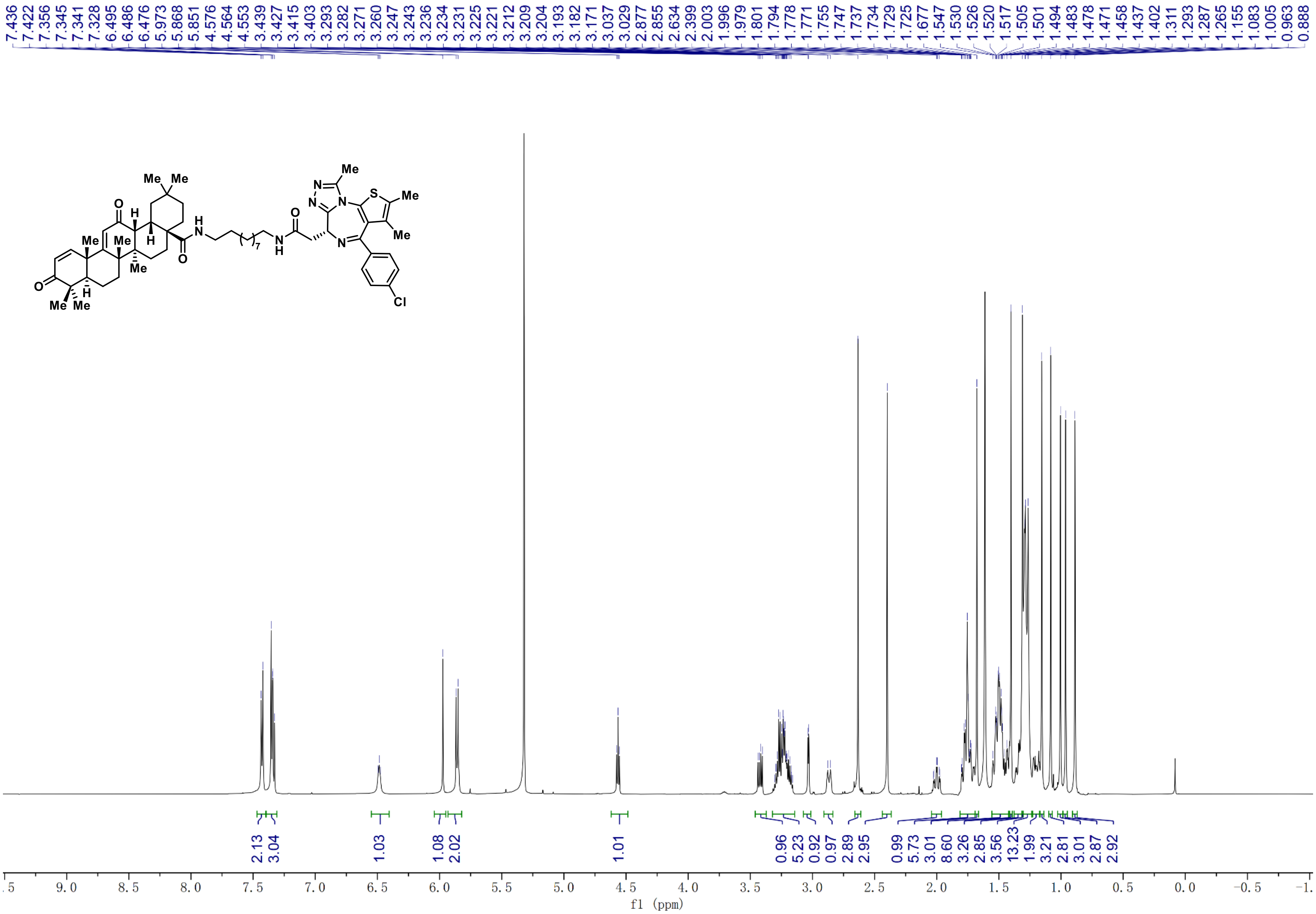

Figure S12. ^{13}C NMR of de-CN-CDDO–JQ1

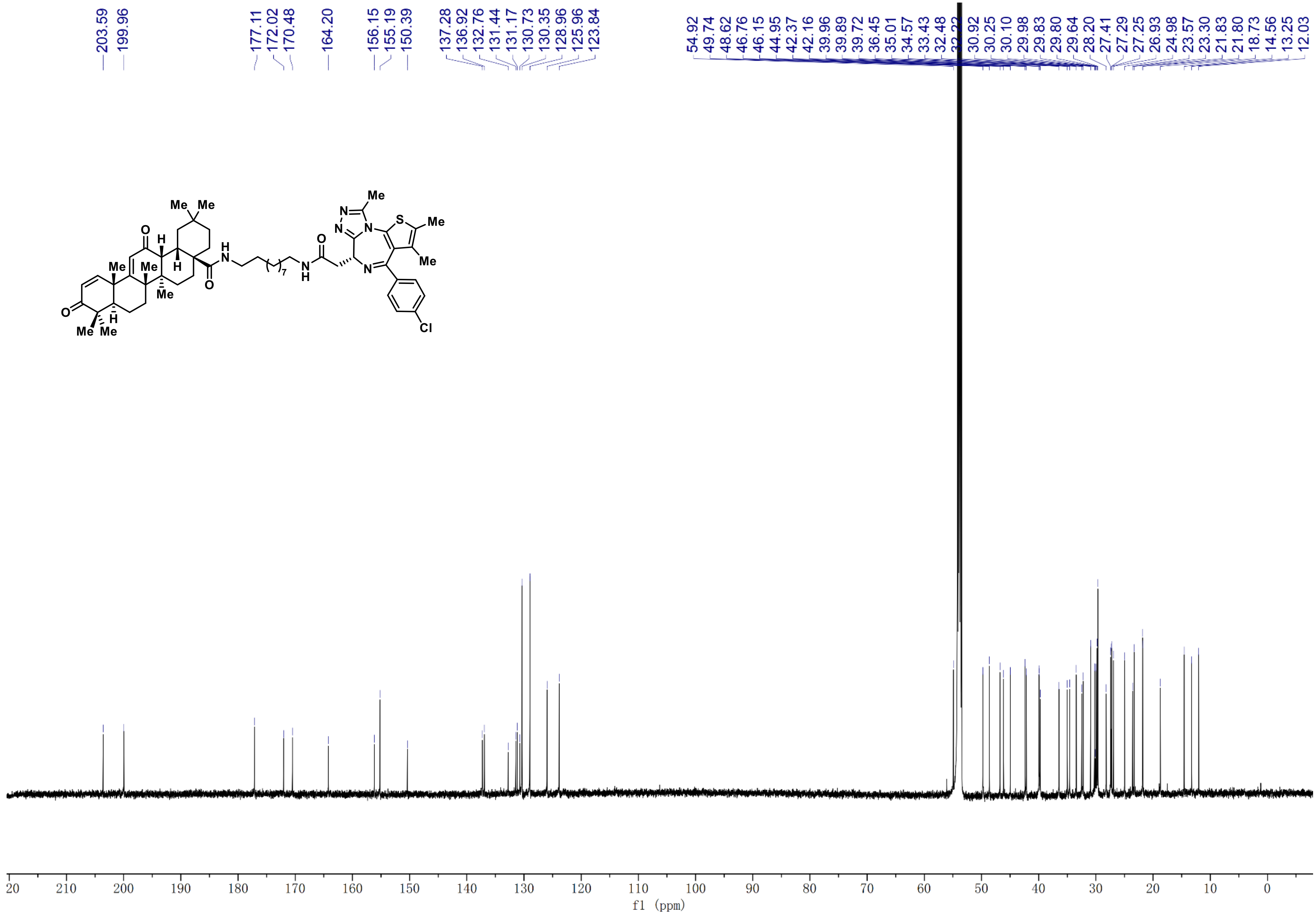

Figure S13. Full length and replicate blots used for manuscript **Figure 2B**

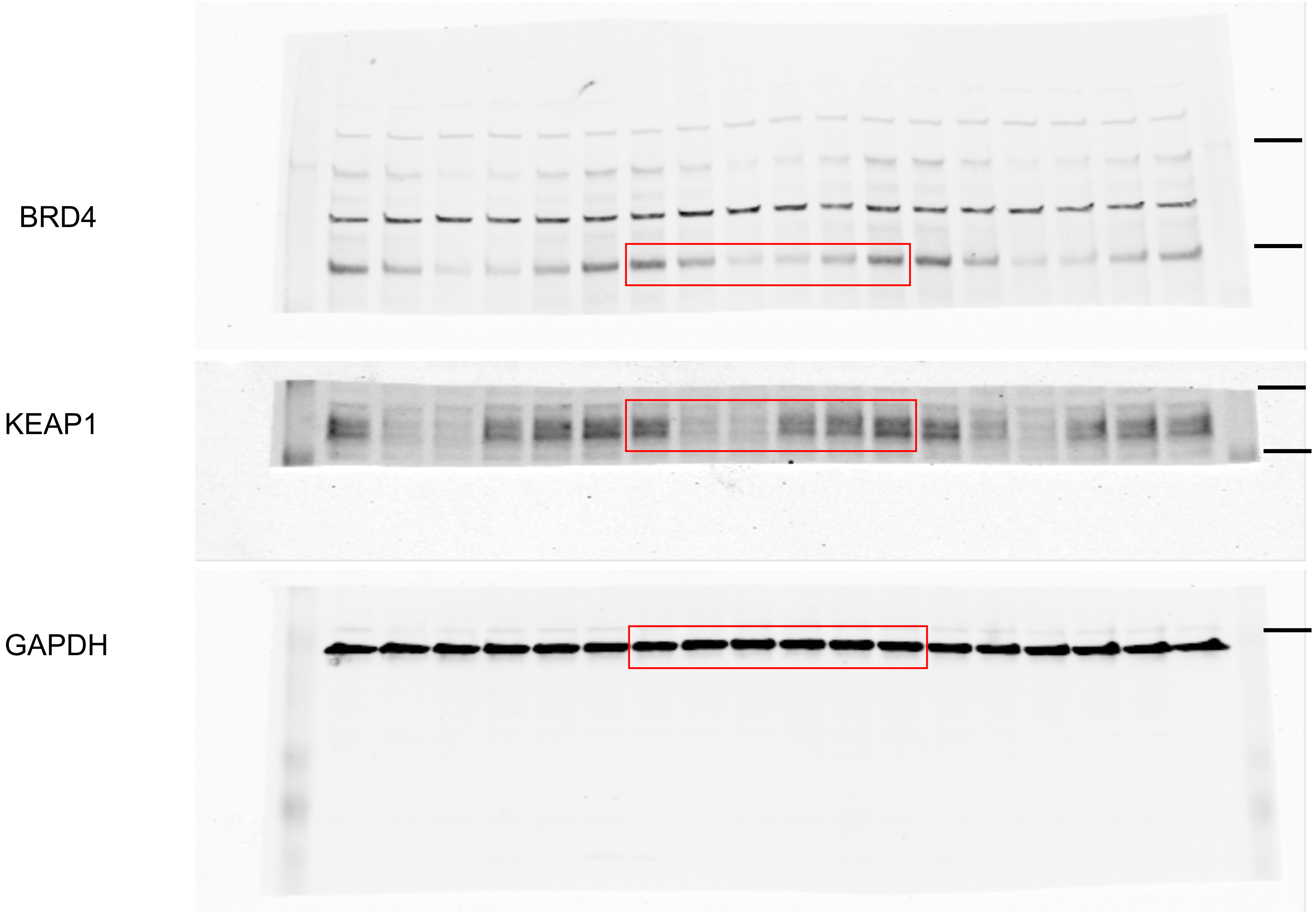

Figure S14. Full length and replicate blots used for manuscript **Figure 2C**

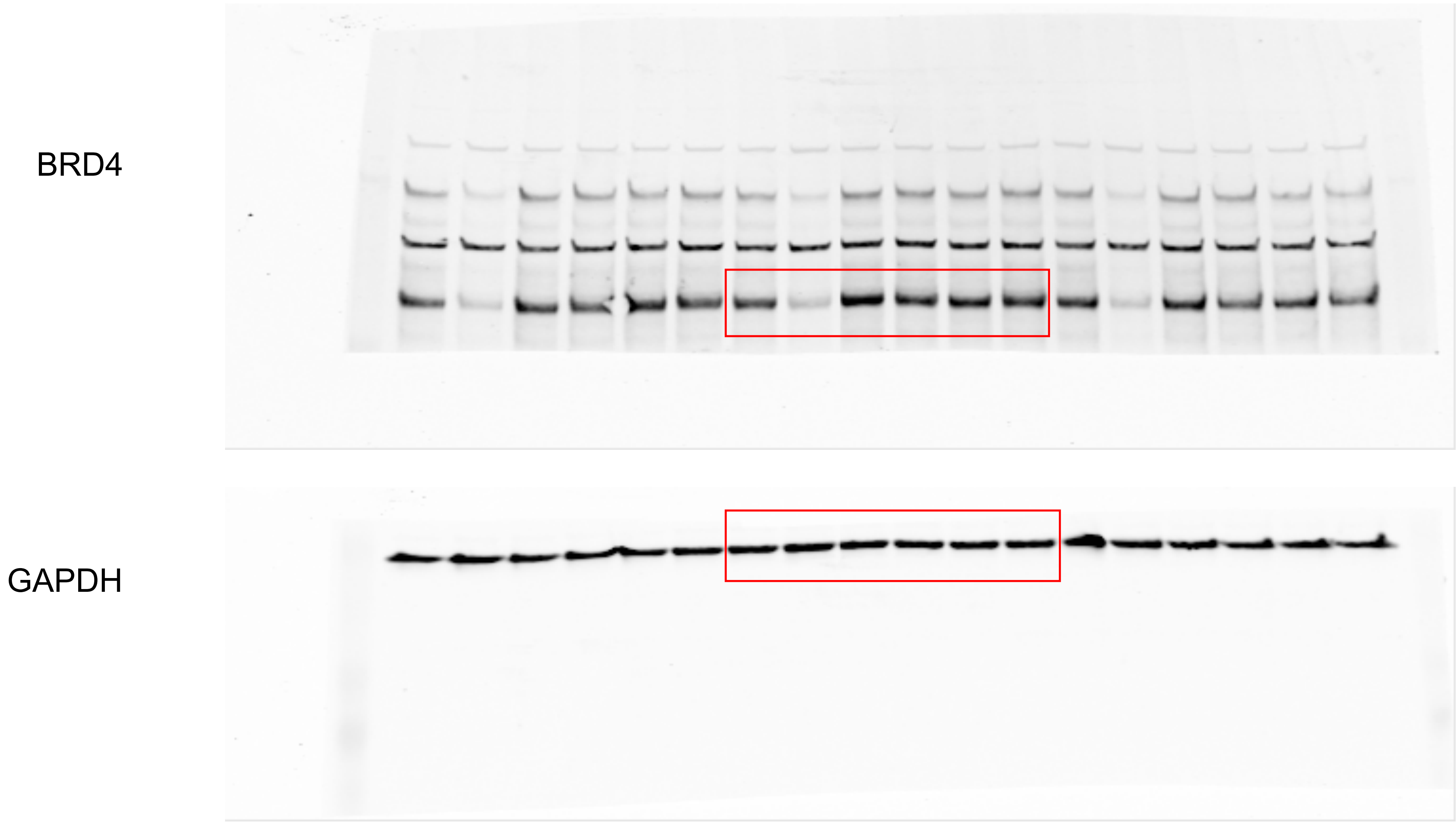

Figure S15. Full length and replicate blots used for manuscript **Figure 2D**

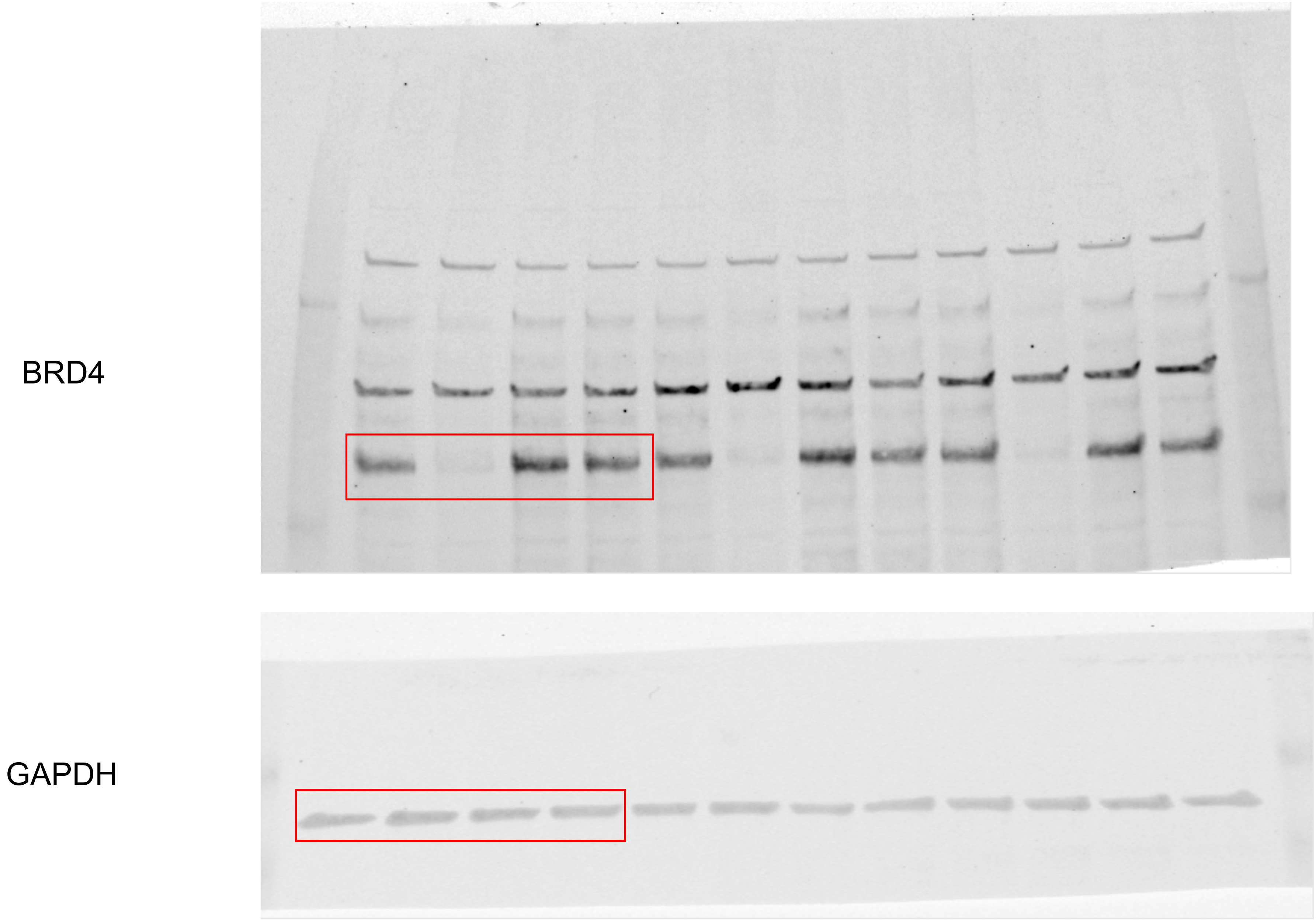

Figure S16. Full length and replicate blots used for manuscript **Figure 3B**

BRD4

KEAP1

GAPDH

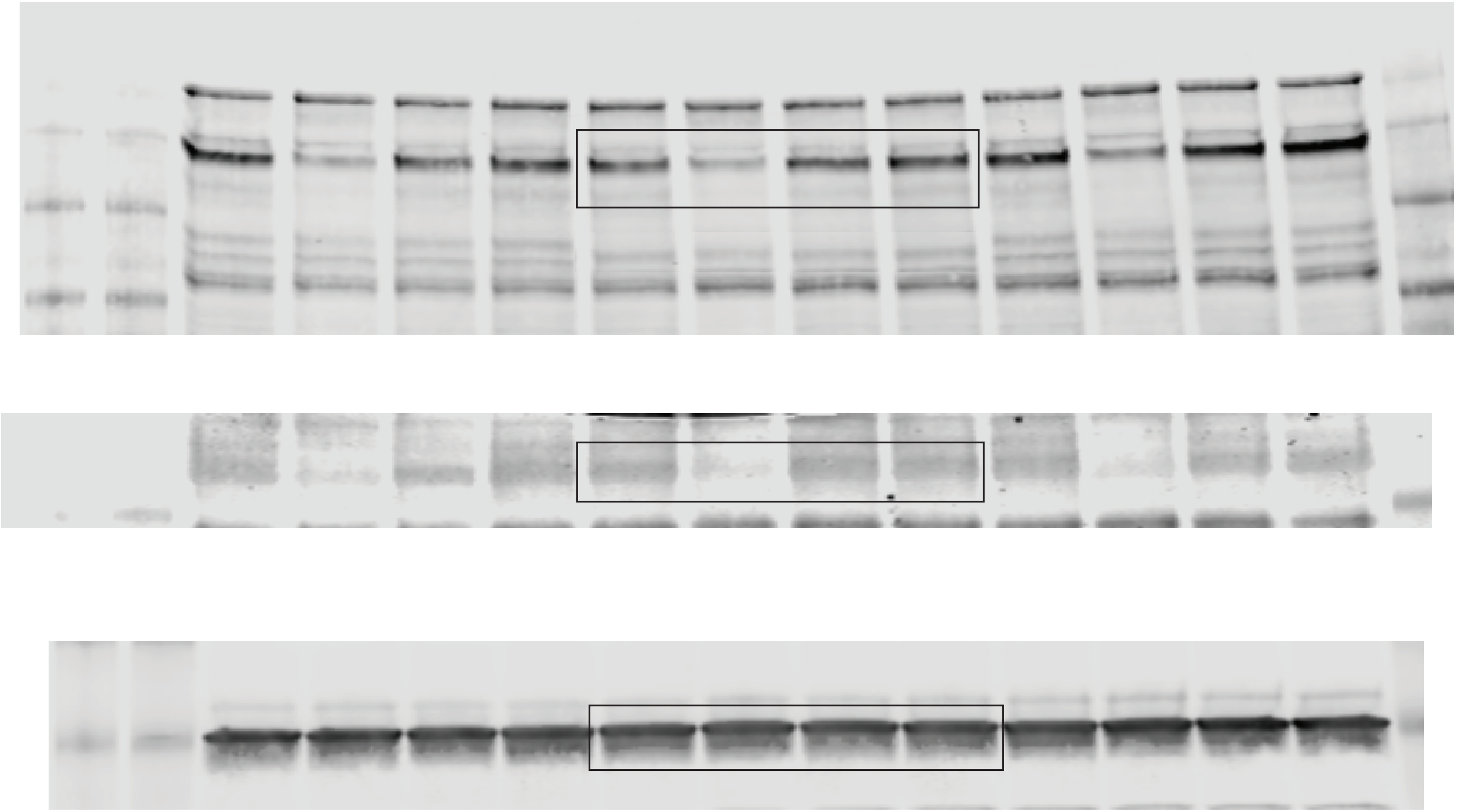

Figure S17. Full length and replicate blots used for manuscript **Figure 4B**

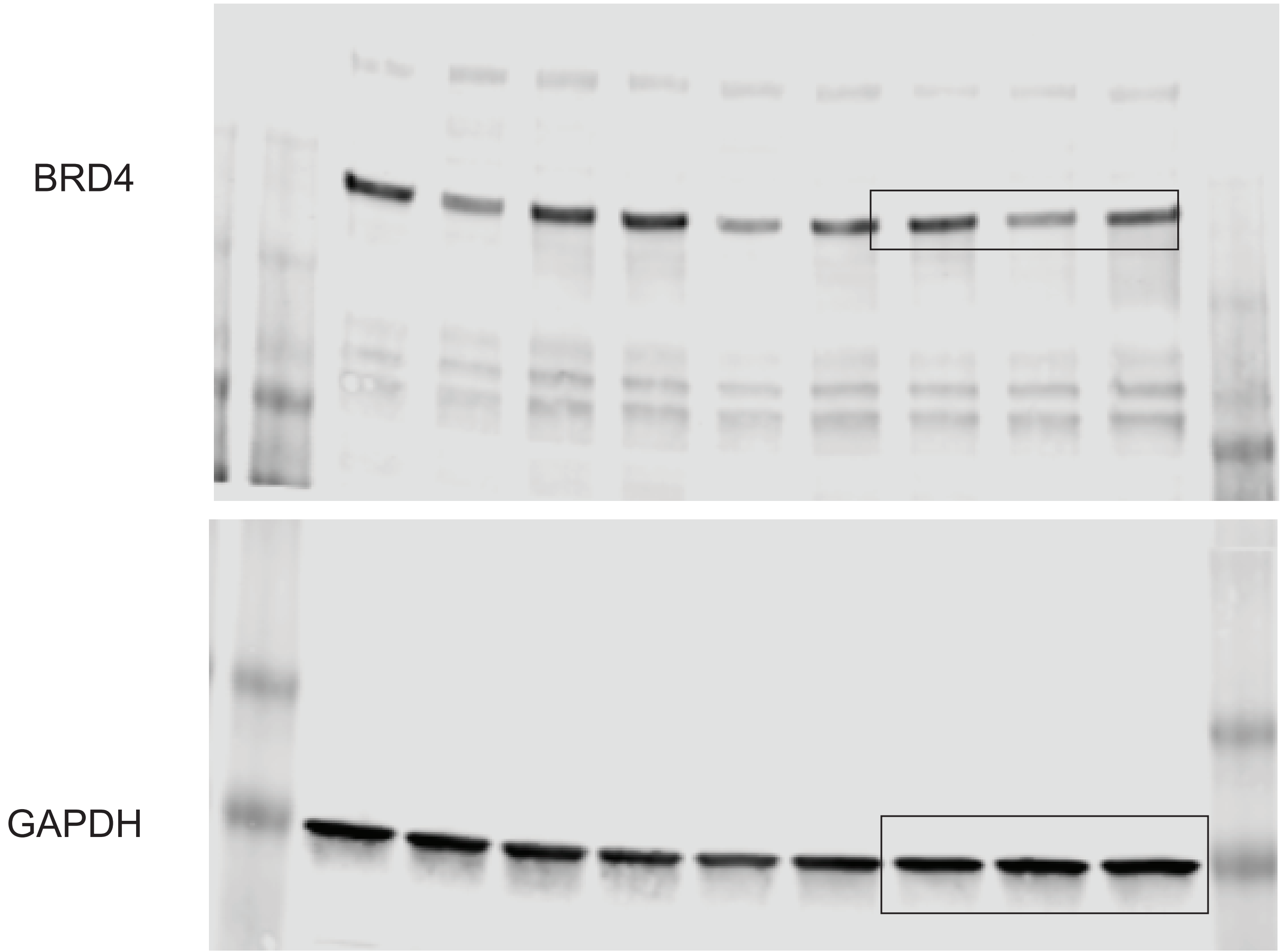

Supplement: Supplementary file 1 — Supplementary Figures. [file 41598_2020_72491_MOESM1_ESM.pdf]
